# Rejection or support? research on labor participation strategy of older adults based on three-party evolutionary game

**DOI:** 10.1371/journal.pone.0346531

**Published:** 2026-04-07

**Authors:** Yan Shen, Yingying Zhong, Fei Sun

**Affiliations:** 1 School of Economics, Management, and Law, Hubei Normal University, Huangshi, Hubei, China; 2 School of Social Work, Michigan State University, East Lansing, Michigan, United States of America; USTC: University of Science and Technology of China, CHINA

## Abstract

To address the demographic challenges posed by accelerated aging and shrinking labor force, it is crucial to develop human resources and encourage labor participation among older adults to achieve active aging. This study constructs a three-party evolutionary game model involving government departments, local enterprises, and older workers based on evolutionary game theory. It analyses the strategic choices of each party during the gaming process and their evolutionary strategies under different conditions, with numerical simulations conducted to examine the impact of parameter adjustments on these evolutionary dynamics. The findings indicate that: the effectiveness of digital government construction serves as a critical determinant for governmental support of older adults’ labor participation; the probability of enterprises actively employing older workers correlates with both the outcomes of corporate digital transformation and labor costs associated with older adults’ employment; older individuals’ likelihood of labor participation relates to employment income and age discrimination, while digital technology empowerment facilitates strategic shifts from negative to positive engagement for both government and enterprises. Based on these conclusions, policy recommendations including strengthening digital government development, accelerating enterprise digital transformation, and fostering age-friendly employment environments are proposed, thereby providing theoretical foundations for implementing national strategies addressing the labor shortage challenges due to population aging.

## 1. Introduction

According to data from China’s National Bureau of Statistics, the population aged 15–59 declined from 916 million to 856 million between 2014 and 2024, with its proportion of the total population decreasing from 67.0% to 60.9%, indicating a marked contraction in the working-age demographic during this period. Meanwhile, the proportion of the population aged 60 and above in China has risen markedly from 15.5% to 22%, surpassing 300 million for the first time and marking the nation’s transition into moderate population aging. Notably, individuals aged 60–69, commonly referred to as the “young-old”, constitute over 55% of this group. This cohort typically possesses accumulated knowledge, extensive experience, and retained professional competencies, constituting a strategically significant form of human capital with considerable potential to support socioeconomic development in the context of demographic transition. A growing body of research also indicates that the employment of older adults does not crowd out job opportunities for younger individuals, which exerts a neutral or even positive effect on the overall labor market performance [1–3]. Under the new demographic norm characterized by low fertility rates, population aging, and regional disparities in population growth and decline, it is necessary to tap into the “second demographic dividend” as a new source of economic growth [4]. These efforts hold significant strategic value for advancing high-quality economic development [5].

Through institutional design and policy innovation, China has gradually established a multi-level framework for developing the human capital of its aging population. The opinions on Strengthening Older Adults Work in the New Era explicitly identify the improvement of policies related to employment, volunteer services, and community governance as key avenues for mobilizing the potential of the “young-old”. The Decision of the CPC Central Committee on Further Deepening Reform and Advancing Chinese-Style Modernization highlights the development of the silver economy and advocates for diversified, tailored employment opportunities for older adults. Concurrently, the State Council’s policy on the Gradual Delay of the Statutory Retirement Age aims to remove legal and institutional barriers to senior labor participation. Despite policy progress, labor force participation among older adults in China remains low. The workforce participation rates for those aged 55–64 in 2020 were 64.7%, 67.9%, and 78.7% in the United States, the United Kingdom, and Japan, respectively, compared with 46.8% in China [6]. These gaps highlight the need for a more effective strategy that integrates institutional design, social support, and policy reform to better mobilize the human capital of the aging population.

The rationality behind labor force participation decisions among older adults is critical to fully realizing the potential of aging-related human capital. This decision-making process entails a dynamic interplay among institutional structures, market incentives, and individual agency. As the primary institutional actor, the government influences older adults’ engagement in the labor market through policy instruments such as gradual retirement reforms, employment regulations, and social protection systems. Employers, guided by market logic, face a trade-off: while aging is associated with declines in physical capacity and learning capacity, older workers often contribute substantial experience and professionalism. These attributes compel firms to balance age-related employment preferences with productivity considerations [7]. At the individual level, participation decisions depend on both internal resources, such as health and skills, and external constraints. Life course theory suggests that preferences regarding work and leisure evolve with age. Young-old individuals with sufficient human capital tend to exhibit stronger employment intentions; however, structural barriers, limited digital literacy, and insufficient incentives frequently inhibit their participation. Accordingly, older adults must navigate the dual pressures of policy constraints and labor market dynamics, making employment decisions that reflect both utility maximization and contextual limitations. This triadic interaction among the government, the market, and older individuals reveals the institutional logic underlying variation in the later-life labor force participation. It offers a valuable perspective on the mechanisms through which retirement reform policies operate and provides an empirical foundation for developing strategies to more effectively mobilize older human capital.

In recent years, there has been growing scholarly interest in applying evolutionary game theory to the study of policy behavior. Researchers have begun to construct a theoretical model that incorporates government participation and enterprise behavior from the logic of micro behavior, aiming to reveal the dynamic game relationship between multiple stakeholders through mathematical modeling and simulation analysis. For example, a large body of literature has studied the dynamic game between corporate environmental pollution and government regulation [8,9], the impact of government participation on corporate green technological innovation [10,11], as well as the issue of big data security in the platform economy [12,13]. Existing research on the decision-making of labor participation behavior of older adults from the three-dimensional perspectives of the government, enterprises, and individuals is relatively dispersed and lacks systematic and in-depth research.

On the basis of existing research, this study constructs a government-enterprise-older adults tripartite evolutionary game model to analyze how government intervention and enterprise behavior jointly affect the employment decision-making process of older adults. It further explores the potential effect of digital technology in enhancing the employment intention of older adults. Unlike previous studies that primarily examine bilateral interactions between the government and enterprises, this paper includes the older adults as rational actors within the game system and introduces digital technology as a key variable in the game matrix, thereby expanding the applicability of the evolutionary game model. The contribution of this paper lies in offering a new theoretical perspective and analytical framework for optimizing the allocation of aging human resources and activating the labor potential of the silver-haired population amid digital transformation.

## 2. Literature review

Existing literature has systematically examined factors contributing to labor participation of older adults across individual, family, and social dimensions. At the individual level, characteristics such as gender, marital status, health condition, education, and wealth level significantly affect labor participation of older adults [14–16]. Among these, both physical health and mental health are widely recognized as key determinants, with better health strongly associated with a higher probability of labor participation [17–19]. From the perspective of family characteristics, intergenerational support networks have a complex and sometimes contradictory impact [20,21]. Intergenerational caregiving, especially grandchild care, is often cited as a constraint on older adults’ nonfarm employment [22], disproportionally affecting grandmothers’ participation in the labor market [23,24]. However, other studies argue that intergenerational caregiving is not significantly related to labor participation in old age [25]. In some contexts, such as among married grandparents in the U.S., caregiving may even motivate continued employment to support younger generations financially [26]. These divergent findings indicate ongoing debate in the field. From the perspective of the social security system, the existing literature examines how institutional arrangements such as pensions and health insurance affect labor participation decisions through economic incentives and risk-sharing mechanisms. Some studies suggest that increased pension income encourages earlier retirement [27], and higher benefits reduce their willingness to remain in the workforce [28]. On the contrary, delaying retirement age and increasing benefits associated with delayed retirement can extend labor force participation of older adults [29–31]. However, these findings are not conclusive. Evidence from China suggests that there is no significant causal relationship between the receipt of the NPS pension and the exit of older adults from the labor market in China, highlighting contextual variability [32].

Traditional game theory has long served as an analytical tool in labor market equilibrium research, explaining the strategic interactions and equilibrium evolution among labor market agents. Models such as the Stackelberg game and the Bertrand game have been used to analyze the entry, exit, and dynamic equilibrium of the labor market [33–35], laying a solid theoretical foundation. More recently, the evolutionary game theory has attracted much attention for its assumption of “bounded rationality”, which better reflects the real-world decision making. Yang and Yan applied an evolutionary game model of firms and employees to explore the strategic choices and evolutionary patterns of firms and employees in human resource management value co-creation in the context of artificial intelligence, finding that cooperative efficiency, organizational performance, and employee satisfaction can be enhanced by optimizing the cost structure and profit-sharing mechanism [36]. Sun et al. demonstrated the facilitating effect of government involvement on vocational training in the construction industry through a game model, concluding that reducing opportunity costs and increasing wage income are key factors to increase the probability of workers’ participation in vocational training [37]. Dong and Yan analyzed the results of the evolutionary game between enterprises and employees on overtime from the perspective of information asymmetry, and found that timely communication and information sharing can effectively promote both parties to achieve Pareto optimality and realize a win-win situation [38]. Some studies have begun to focus on the dynamic game mechanisms of employment for special groups. Dong et al. combined evolutionary game theory and system dynamics to analyze the evolutionary game process of older adults’ bridge employment between the government and enterprises, pointing out that the enthusiasm of enterprises in absorbing the employment of older adults is significantly increased under appropriate dynamic reward and punishment mechanisms [39]. Similarly, Sun et al. constructed a tripartite evolutionary game model involving the government, employers, and persons with disabilities, and the findings indicate that active government guidance and enhancing workers’ skills can significantly increase the labor participation of persons with disabilities [40].

Beyond labor market participation, multi-actor evolutionary models are increasingly used to address governance challenges in AI regulation [41,42], environmental monitoring [43], healthcare systems [44], and energy policy [45]. Across these applications, information transparency and incentive alignment among stakeholders are commonly identified as key drivers of collective stability. While many of these studies employ stochastic dynamics to capture finite-population effects, they offer a complementary perspective to the deterministic evolutionary framework adopted here. Despite such progress in diverse governance fields, most existing research on labor participation still focuses on bilateral relationships, such as interactions between governments and enterprises or between enterprises and employees. Few studies have developed a unified analytic framework that simultaneously incorporates governments, enterprises, and workers. Moreover, limited attention has been paid to the distinctive employment behaviors of older workers compared to the general workforce, which restricts a comprehensive understanding of the mechanisms influencing labor participation among older adults. To address this gap, this study constructs a tripartite evolutionary game model capturing the interactions among government, enterprises, and older workers, with particular attention to how digital technology and policy interventions jointly shape these evolutionary dynamics.

## 3. Assumptions

This study involves three types of agents: government departments, local enterprises, and older workers. It is assumed that all three parties in the game are bounded rational individuals. Government departments aim to realize social interests, while local enterprises and older workers aim to maximize their interests. Additionally, they do not fully understand each other’s strategies or utility functions. Strategy adjustment therefore occurs dynamically until a stable evolutionary outcome is reached.

To translate these interactions into a transparent and tractable mathematical framework, the tripartite evolutionary game is parameterized to reflect the incentives and constraints that are directly relevant to each party’s decision-making. Although multiple parameters are introduced to capture key features of elderly employment in a digitalized context (e.g., digital efficiency and age-related barriers), each parameter has a clearly defined functional role. Importantly, parameters do not enter the replicator dynamics system as isolated absolute values; instead, strategic incentives are governed by relative payoff differences (e.g., Δπ=Benefit−Cost). Consequently, the existence and location of stable equilibria are determined by the relationships among payoff components, rather than by the absolute magnitudes of individual parameters. This structure keeps the model analytically tractable while preserving mechanism-level interpretability.

Based on the above considerations, the following assumptions are introduced to analyze the evolutionary dynamics among the three parties.

Assumption one: Each participant has two alternative strategies. For government departments, their strategic portfolio is $\alpha =\left\{\alpha_{\text{1}},\alpha_{\text{2}}\right\}$, where $\alpha_{\text{1}}$ represents positive action, including publicizing and guiding the employment of older adults, as well as regulating whether enterprises have age discrimination in employment, etc., $\alpha_{\text{2}}$ represents negative action. The probability of choosing positive action is x (0≤x≤1), while the probability of choosing negative action is (1−x). For local enterprises, their strategic portfolio is $\beta =\left\{\beta_{\text{1}},\beta_{\text{2}}\right\}$, where $\beta_{\text{1}}$ is positively hiring older adults, which includes encouraging employees to delay retirement and providing re-employment positions for them, etc., and $\beta_{\text{2}}$ is negatively employing older adults. The probability of positively employing older adults is y (0≤y≤1), and the probability of negatively hiring older adults is (1−y). For older adults, their strategic portfolio is $\gamma =\left\{\gamma_{\text{1}},\gamma_{\text{2}}\right\}$, where $\gamma_{\text{1}}$ refers to delayed retirement or re-employment, and $\gamma_{\text{2}}$ refers to not participating in the labor force. The probability of choosing $\gamma_{\text{1}}$ is z (0≤z≤1), while the probability of choosing $\gamma_{\text{2}}$ is (1−z).

Assumption two: Gains and losses for government departments. For government departments, strategic incentives are primarily governed by the net cost differential between positive and negative actions, together with the net social externality associated with elderly employment. The revenue of the government departments is R1. Under the government sector’s positive strategy, it is necessary to publicize and guide social trends, such as promoting older adults’ labor participation and regulating employers against age discrimination, which incurs a cost of C1. Since the Ministry of Human Resources and Social Security explicitly resists age discrimination in employment, when an enterprise refuses to hire older adults despite their willingness to work, a penalty F will be imposed on the enterprise by the government departments, and the complainant will receive the fine. If older adults do not wish to be employed, the fine will become the government’s forfeiture revenue. Under this strategy, labor participation among older adults can effectively promote the development of older adults’ manpower resources and alleviate the shortage of the working-age labor force, which can generate additional social benefits U1. Under the government’s negative strategy, the cost of regulation is C2 (C1>C2).

Assumption three: Gains and losses for the local enterprises. For local enterprises, hiring incentives are primarily determined by the relative labor cost differential between employing older workers and alternative labor, together with net reputational/regulatory payoffs. The revenue of the local enterprise is R3. Under the enterprise’s strategy of positively employing older adults, the labor cost is C3. Experienced older employees will bring additional benefits to the enterprise, and a good employment environment enhances the enterprise’s social reputation, resulting in an extra benefit U2. Under the enterprise’s strategy of negatively employing older adults, the replacement cost of re-recruiting and retraining other laborers is C4. This strategy will diminish the enterprise’s social reputation, leading to a loss L2.

Assumption four: Gains and losses for older adults. For older adults, participation decisions are governed by the net utility difference between labor participation and non-participation. When older adults choose to delay retirement or re-employment, they not only gain wage income and welfare benefits but also improve communication with social groups outside their family, leading to benefits such as social identity and mental health improvement [46], which produces revenue R5. At the same time, they must incur a certain amount of employment cost C5. If enterprises choose to recruit older adults at this time negatively, it will have detrimental effects on their mental health due to age discrimination, resulting in a loss L3. Under the strategy of opting for non-labor participation, older adults can enjoy the benefits of leisure or household care, generating revenue R6, while incurring the opportunity cost of not being employed C6 (R5>R6>C6>C5). This strategy increases older adults’ choice of non-participation in the labor market, further putting pressure on the government’s pension finances, which can result in a loss L1.

Assumption five: Through government process rebuilding and data sharing, the digital transformation of government systems can significantly increase the transparency and efficiency of government departments [47], reduce the cost of administrative approvals, and enhance the ability to provide public services. Therefore, it is assumed that the distribution coefficient of the digital government construction between the benefit and cost parameters of government departments is r under the strategy of positive action. Data-driven innovation contributes to total factor productivity in enterprises and replaces inefficient aspects of traditional factors of production, systematically reducing labor costs and operating expenses for the enterprise. Thus, it is assumed that the coefficient of distribution of the enterprise’s digital transformation between its benefit and cost parameters is s under the strategy of positively employing older adults. The development of digital technology and digital economy expands the spatio-temporal boundaries of employment [48], generates a significant employment promotion effect on the aging labor market through industrial structure upgrades and the derivation of emerging occupations, and decreases the cost of skills training for older adults. Therefore, it is assumed that the distribution coefficient of digital technology between the benefit and cost parameters of older adults is t under the strategy of labor participation.

The above specific parameters and their meanings are shown in Table 1.

**Table 1:** Parameters and meaning of game behavior.

Parameters	Meaning
x	Probability of a government department choosing a positive action
y	Probability of a local enterprise choosing to positively employ older adults
z	Probability of older adults choosing delayed retirement or re-employment
R1	Revenue of the government department
C1	Cost of choosing positive action for a government department
C2	Cost of choosing a negative action for a government department
F	Penalty of negatively employing older adults for local enterprises
U1	An additional benefit of choosing positive action for a government department
L1	The social burden of older adults choosing non-labor participation
R3	Revenue of the local enterprise
C3	Labor cost of older adults
C4	Alternative labor cost
U2	Benefits of choosing positively employing older adults for local enterprises
L2	Loss of choosing negatively employing older adults for local enterprise
R5	Benefits of labor participation for older adults
C5	Cost of labor participation for older adults
L3	Loss for older adults due to age discrimination
R6	Benefits of non-labor participation for older adults
C6	Opportunity cost of non-labor participation for older adults
r	Government digitalization efficacy coefficient
s	Enterprise digital transformation efficacy coefficient
t	Gerontological digital inclusion coefficient

Given the above assumptions, a tripartite evolutionary game model is constructed to explain older adults’ labor participation considering the influence of governmental departments, local enterprises, and older adults. The evolutionary game payoff matrix is shown in Table 2.

**Table 2:** Payoff matrix of three actors.

Game participants		Local enterprise	Older adults	
			**Labor participation** (z)	**Non-labor participation** (1−z)
Government department	Positive action (x)	Positively hiring older adults (y)	(1+r)R1−(1−r)C1+U1	(1+r)R1−(1−r)C1−L1
			(1+s)R3−(1−s)C3+U2 (1+t)R5−(1−t)C5	(1+s)R3−(1−s)C4 R6−C6
		Negatively hiring older adults (1−y)	(1+r)R1−(1−r)C1+U1 R3−C4−F	(1+r)R1−(1−r)C1−L1+F
			(1+t)R5−(1−t)C5+F−L3	R3−C4−L2−F R6−C6
	Negative action (1−x)	Positively hiring older adults (y)	R1−C2 (1+s)R3−(1−s)C3+U2 (1+t)R5−(1−t)C5	R1−C2−L1 (1+s)R3−(1−s)C4 R6−C6
		Negatively hiring older adults (1−y)	R1−C2	R1−C2−L1
			R3−C4	R3−C4
			(1+t)R5−(1−t)C5−L3	R6−C6

When government departments choose to take positive action, local enterprises choose to positively employ older adults, and older adults choose to participate in labor, the benefits for government departments and local enterprises are their respective revenues minus the cost of positive action, plus the resulting additional revenues, as well as the impacts brought by government digitalization and enterprise digital transformation. That is, (1+r)R1−(1−r)C1+U1 for government departments and (1+s)R3−(1−s)C3+U2 for local enterprises. The benefit for older adults is the revenue from labor participation minus the cost, along with the impact brought by the gerontological inclusion coefficient, i.e., (1+t)R5−(1−t)C5. When government departments choose positive action, local enterprises choose to employ older adults negatively, and older adults choose labor participation, the benefit for government departments remains unchanged. Under the active supervision of government departments, fines will be imposed on enterprises that employ older adults negatively and used to compensate the older adults. Under this strategy, the benefit for local enterprises is revenue minus the cost of alternative employment and the fine, i.e., R3−C4−F. The benefit for older adults additionally includes subtracting the loss due to age discrimination and adding the compensation received, i.e., (1+t)R5−(1−t)C5+F−L3. When government departments choose negative action, local enterprises choose to positively employ older adults, and older adults choose labor participation, the benefits for local enterprises and older adults are the same as in the first strategy. The benefit for government departments is revenue minus the cost of negative action, i.e., R1−C2. When government departments choose negative action, local enterprises choose to employ older adults negatively, and older adults choose labor participation, no fines are generated at this time, and only the older adults suffer losses from age discrimination. Therefore, the benefits for government departments and local enterprises are revenue minus the cost of negative action, i.e., R1−C2 and R3−C4, respectively. The benefit for older adults is (1+t)R5−(1−t)C5−L3.

When older adults choose non-labor participation, regardless of the strategies of government departments and local enterprises, the benefit for older adults is the revenue from non-labor participation minus the opportunity cost, i.e., R6−C6. When older adults choose non-labor participation, government departments will incur fiscal burdens, and local enterprises can only employ non-older adults. If both government departments and local enterprises choose positive action, the benefit for government departments is revenue minus the cost of positive action, the fiscal burden loss, and the impact of government digitalization, i.e., (1+r)R1−(1−r)C1−L1. The benefit for local enterprises is revenue minus the cost of alternative employment and the impact of enterprise digital transformation, i.e., (1+s)R3−(1−s)C4. If government departments choose positive action while local enterprises choose negative action, fines will be generated under the supervision of government departments, but these fines cannot be compensated to the older adults and will instead serve as additional revenue for government departments. Therefore, the revenue of government departments will additionally include the fines, i.e., (1+r)R1−(1−r)C1−L1+F. Local enterprises will face losses in social reputation, so their benefit is R3−C4−L2−F. When government departments choose negative action, no fines will be generated, and there will be no impact on the reputation of local enterprises. Therefore, the benefit for government departments is revenue minus the cost of negative action and the fiscal burden, i.e., R1−C2−L1. Under this strategy, the benefit for local enterprises positively employing older adults is (1+s)R3−(1−s)C4, and the benefit for negatively employing older adults is R3−C4.

## 4. Model analysis

In evolutionary games, enterprises, governments, and individuals are all decision-makers of bounded rationality; the players in the game usually cannot find the optimal strategy immediately at the initial stage. Instead, they learn the game in the process and find the optimal strategy through trial and error. Therefore, equilibrium is not the result of a one-time choice but is in a process of continuous adjustment and improvement. Even if equilibrium is achieved, deviations may occur again [49]. The “replication dynamics” mechanism of biological evolution can simulate the learning and dynamic adjustment process of the game players quite well, while the “evolutionary stability strategy” applies to analyzing the stability and dynamic development trends during the learning and adjustment process. Therefore, the replication dynamic equations of behavioral strategies for enterprises, governments, and older adults are constructed respectively below, and their evolutionary stability strategies are analyzed. Additionally, to further examine the robustness of the evolutionary outcomes under stochastic perturbations, a stochastic evolutionary extension of the model is provided in S1 File.

### 4.1. Analysis of the stability of the government department strategy

The expected benefits of choosing positive action or negative action in government department, as well as the average expected benefits are:


$$\left\{ \begin{array}{c} E_{x\text{1}}=\;yz\left((\text{1}+r)R\text{1}-(\text{1}-r)C\text{1}+U\text{1}\right)+y(\text{1}-z)\left((\text{1}+r)R\text{1}-(\text{1}-r)C\text{1}-L\text{1}\right)+z(\text{1}-y)\\ \left((\text{1}+r)R\text{1}-(\text{1}-r)C\text{1}+U\text{1})+(\text{1}-y)(\text{1}-z)((\text{1}+r)R\text{1}-(\text{1}-r)C\text{1}-L\text{1}+F\right)\\ E_{x\text{2}}=\;yz(R\text{1}-C\text{2})+y(\text{1}-z)(R\text{1}-C\text{2}-L\text{1})+z(\text{1}-y)(R\text{1}-C\text{2})+(\text{1}-y)(\text{1}-z)(R\text{1}-C\text{2}-L\text{1})\\ \overset{―}{E_{x}}=xE_{x\text{1}}+(\text{1}-x)E_{x\text{2}} \end{array}\right.\;$$

The replication dynamics equation for the government department is. $F(x)=\frac{dx}{dt}=x(\text{1}-x)[rR\text{1}-(\text{1}-r)C\text{1}+C\text{2}+zU\text{1}+(y-\text{1})(z-\text{1})F]$

The first-order derivatives of x and the set G(y) are:


$$\frac{dF(x)}{dx}=(\text{1}-\text{2}x)\left[rR\text{1}-(\text{1}-r)C\text{1}+C\text{2}+zU\text{1}+(y-\text{1})(z-\text{1})F\right]$$

G(y)=rR1−(1−r)C1+C2+zU1+(y−1)(z−1)F

**Fig 1:**
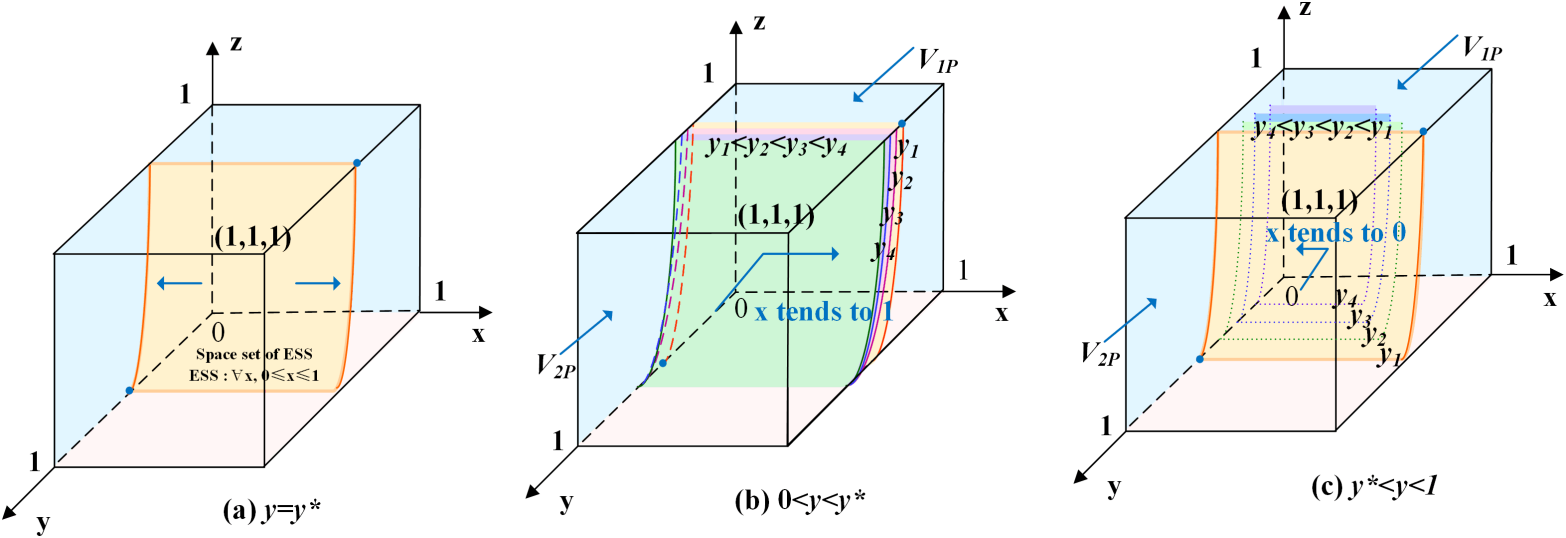
Phase diagram of government department’s strategy.

According to the stability theorem of differential equations, the following conditions must be satisfied for a government department’s positive action strategy to be in an equilibrium stable state: F(x)=0 and $\frac{dF(x)}{dx}<\text{0}$. Since $\frac{\partial G(y)}{\partial y}<\text{0}$, G(y) is a decreasing function with respect to y. Thus, when $y=\frac{rR\text{1}-(\text{1}-r)C\text{1}+C\text{2}+zU\text{1}+(\text{1}-z)F}{(\text{1}-z)F}=y^{*}$, G(y)=0, at which point $\frac{dF(x)}{dx}\equiv \text{0}$, the government department cannot determine a stabilization strategy. When $y<y^{*}$, G(y)>0, then x=1 is the evolutionary stabilization strategy for government department. On the contrary, when $y>y^{*}$, G(y)<0, then x=0 is the evolutionary stabilization strategy for government department. The related phase diagram is shown in Fig 1.

Fig 1 shows the volume $V_{\text{1}P}$ of the probability that government department choosing positive action, and the volume $V_{\text{2}P}$ of the probability that government department choosing negative action. When $y<y^{*}$, as shown in Fig 1(b) where $y_{\text{1}}<y_{\text{2}}<y_{\text{3}}<y_{\text{4}}$, the volume of $V_{\text{1}P}$ gradually increases, the volume of $V_{\text{2}P}$ gradually decreases, x gradually approaches 1, and the probability of the government department choosing positive action increases. When $y>y^{*}$, as shown in Fig 1(c) where $y_{\text{4}}<y_{\text{3}}<y_{\text{2}}<y_{\text{1}}$, the volume of $V_{\text{1}P}$ gradually decreases, the volume of $V_{\text{2}P}$ gradually increases, x gradually approaches 0, and the probability of the government department choosing negative action increases.

The expressions of $V_{\text{1}P}$ and $V_{\text{2}P}$ can be calculated by the following:


$$V_{\text{1}P}=\int_{\text{0}}^{\text{1}}\int_{\text{0}}^{\frac{rR\text{1}-(\text{1}-r)C\text{1}+C\text{2}+F}{F-U\text{1}}}\frac{rR\text{1}-(\text{1}-r)C\text{1}+C\text{2}+zU\text{1}+(\text{1}-z)F}{(\text{1}-z)F}\;dzdx$$

$$V_{\text{2}P}=\text{1}-V_{\text{1}P}$$

Inference one: The probability of government department choosing positive action is positively correlated with the revenue of government department, the costs of choosing negative action, the additional benefit of choosing positive action, and the government digitalization efficacy coefficient, and negatively correlated with the costs of choosing positive action.

Proof: Since $\frac{\partial V_{\text{1}P}}{\partial R\text{1}}>\text{0}$, $\frac{\partial V_{\text{1}P}}{\partial C\text{2}}>\text{0}$, $\frac{\partial V_{\text{1}P}}{\partial r}>\text{0}$, $\frac{\partial V_{\text{1}P}}{\partial U\text{1}}>\text{0}$, $\frac{\partial V_{\text{1}P}}{\partial C\text{1}}<\text{0}$, when R1, C2, r, and U1 increase, the volume of $V_{\text{1}P}$ grows, and the probability of government department choosing positive action increases at this time. When C1 increases, the volume of $V_{\text{2}P}$ grows, and the probability of government department choosing negative action increases.

Inference one suggests that the greater the government digitalization efficacy coefficient, the more likely it is to induce a choice of positive action. There is a substitution of the regulatory costs of positive and negative action; when the costs of positive regulation are higher, the government is more likely to choose negative action.

### 4.2 Analysis of the stability of the local enterprise strategy

The expected benefits of choosing positively hiring older adults or negatively hiring older adults in local enterprise, as well as the average expected benefits are:


$$\left\{ \begin{array}{c} E_{y\text{1}}=xz\left((\text{1}+s)R\text{3}-(\text{1}-s)C\text{3}+U\text{2}\right)+x(\text{1}-z)\left((\text{1}+s)R\text{3}-(\text{1}-s)C\text{4}\right)+z(\text{1}-x)\\ \left((\text{1}+s)R\text{3}-(\text{1}-s)C\text{3}+U\text{2})+(\text{1}-x)(\text{1}-z)((\text{1}+s)R\text{3}-(\text{1}-s)C\text{4}\right)\\ E_{y\text{2}}=\;xz(R\text{3}-C\text{4}-F)+x(\text{1}-z)(\;R\text{3}-C\text{4}-F-L\text{2})+z(\text{1}-x)(R\text{3}-C\text{4})+(\text{1}-x)(\text{1}-z)(R\text{3}-C\text{4})\\ \overset{―}{E_{y}}=yE_{y\text{1}}+(\text{1}-y)E_{y\text{2}} \end{array}\right.\;$$

The replication dynamics equation for the local enterprise is. $F(y)=\frac{dy}{dt}=y(y-\text{1})[z(\text{1}-s)(C\text{3}-C\text{4})-s(R\text{3}+C\text{4})-xF-zU\text{2}-x(\text{1}-z)L\text{2}]$

The first-order derivatives of y and the set G(x) are:


$$\frac{dF(y)}{dy}=(\text{2}y-\text{1})\left[z(\text{1}-s)(C\text{3}-C\text{4})-s(R\text{3}+C\text{4})-xF-zU\text{2}-x(\text{1}-z)L\text{2}\right]$$

G(x)=z(1−s)(C3−C4)−s(R3+C4)−xF−zU2−x(1−z)L2

**Fig 2:**
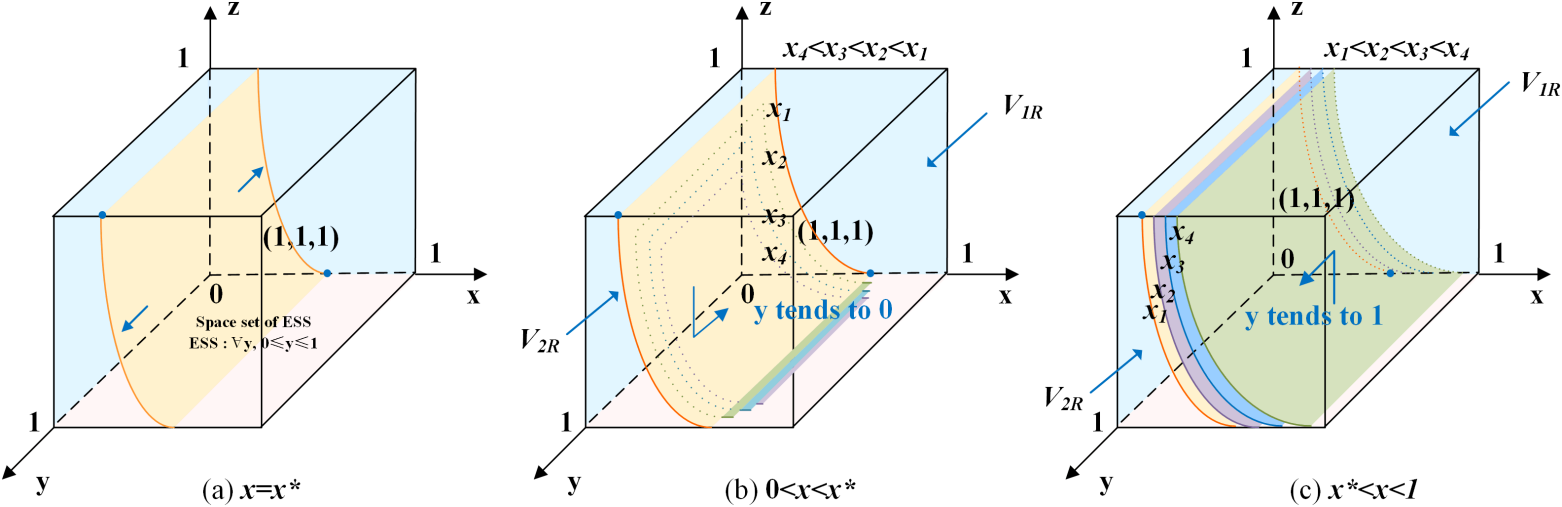
Phase diagram of local enterprise’s strategy.

According to the stability theorem of differential equations, the following conditions must be satisfied for local enterprise’s choice of positively hiring older adults to be in an equilibrium stable state: F(y)=0 and $\frac{dF(y)}{dy}<\text{0}$. Since $\frac{\partial G(x)}{\partial x}<\text{0}$, G(x) is a decreasing function with respect to x. Thus, when $x=\frac{z(\text{1}-s)(C\text{3}-C\text{4})-s(R\text{3}+C\text{4})-zU\text{2}}{F+(\text{1}-z)L\text{2}}=x^{*}$, G(x)=0, at which point $\frac{dF(y)}{dy}\equiv \text{0}$, the local enterprise cannot determine a stabilization strategy. When $x<x^{*}$, G(x)>0, then y=0 is the evolutionary stabilization strategy for local enterprise. On the contrary, when $x>x^{*}$, G(x)<0, then y=1 is the evolutionary stabilization strategy for local enterprise. The related phase diagram is shown in Fig 2.

Fig 2 demonstrates the volume $V_{\text{2}R}$ of the probability that local enterprise choosing positively hiring older adults, and the volume $V_{\text{1}R}$ of the probability that local enterprise choosing negatively hiring older adults. When $x<x^{*}$, as shown in Fig 2(b) where $x_{\text{4}}<x_{\text{3}}<x_{\text{2}}<x_{\text{1}}$, the volume of $V_{\text{1}R}$ gradually increases, the volume of $V_{\text{2}R}$ gradually decreases, y gradually approaches 0, and the probability of the local enterprise choosing negatively hiring older adults increases. When $x>x^{*}$, as shown in Fig 2(c) where $x_{\text{1}}<x_{\text{2}}<x_{\text{3}}<x_{\text{4}}$, the volume of $V_{\text{1}R}$ gradually decreases, the volume of $V_{\text{2}R}$ gradually increases, y gradually approaches 1, and the probability of the local enterprise choosing positively hiring older adults increases.

The expressions of $V_{\text{1}R}$ and $V_{\text{2}R}$ can be calculated by the following:


$$V_{\text{1}R}=\int_{\text{0}}^{\text{1}}\int_{\text{0}}^{\text{1}}\frac{z(\text{1}-s)(C\text{3}-C\text{4})-s(R\text{3}+C\text{4})-zU\text{2}}{F+(\text{1}-z)L\text{2}}dzdy$$

$$V_{\text{2}R}=\text{1}-V_{\text{1}R}$$

Inference two: The probability of local enterprise choosing positively hiring older adults is positively correlated with the revenue of local enterprise, the alternative labor cost, the benefit of choosing positively hiring older adults, the loss of choosing negatively hiring older adults, penalty of negatively hiring older adults, and the enterprise digital transformation efficacy coefficient, and negatively correlated with the labor cost of older adults.

Proof: Since $\frac{\partial V_{\text{2}R}}{\partial R\text{3}}>\text{0}$, $\frac{\partial V_{\text{2}R}}{\partial C\text{4}}>\text{0}$, $\frac{\partial V_{\text{2}R}}{\partial U\text{2}}>\text{0}$, $\frac{\partial V_{\text{2}R}}{\partial L\text{2}}>\text{0}$, $\frac{\partial V_{\text{2}R}}{\partial F}>\text{0}$, $\frac{\partial V_{\text{2}R}}{\partial s}>\text{0}$, $\frac{\partial V_{\text{2}R}}{\partial C\text{3}}<\text{0}$, when R3, C4, U2, L2, F and s increase, the volume of $V_{\text{2}R}$ grows, and the probability of local enterprise choosing positively hiring older adults increases at this time. When C3 increases, the volume of $V_{\text{1}R}$ grows, and the probability of local enterprise choosing negatively hiring older adults increases.

Inference two suggests that the greater the additional gains of enterprise digital transformation and positively hiring older adults, the easier it is for the enterprise to choose positively hiring older adults. There is substitution of the labor costs of positive and negative action, when the costs of alternative labor are higher, the more likely the enterprise is to make the choice of positively hiring older adults. Negative action will lead to damage of the enterprise’s social reputation, reduction of intangible assets, and punishment by government departments, which can be regarded as the opportunity cost incurred by the enterprise when choosing to negatively hire older adults, so the punishment of the government departments on the enterprise should be appropriately set in order to achieve the ideal stable state.

### 4.3. Analysis of the stability of the older adults strategy

The expected benefits of older adults choosing labor participation or non labor participation, as well as the average expected benefits are:

$\left\{ \begin{array}{c} E_{Z\text{1}}=xy\left((\text{1}+t)R\text{5}-(\text{1}-t)C\text{5}\right)+x(\text{1}-y)\left((\text{1}+t)R\text{5}-(\text{1}-t)C\text{5}+F-L\text{3}\right)+y(\text{1}-x)\\ \left((\text{1}+t)R\text{5}-(\text{1}-t)C\text{5})+(\text{1}-x)(\text{1}-y)((\text{1}+t)R\text{5}-(\text{1}-t)C\text{5}-L\text{3}\right)\\ E_{Z\text{2}}=\;xy(R\text{6}-C\text{6})+x(\text{1}-y)(R\text{6}-C\text{6})+y(\text{1}-x)(R\text{6}-C\text{6})+(\text{1}-y)(\text{1}-x)(R\text{6}-C\text{6})\\ \overset{―}{E_{z}}=zE_{z\text{1}}+(\text{1}-z)E_{z\text{2}} \end{array}\right.\;$

The replication dynamics equation for the older adults is:


$$F(z)=\frac{dz}{dt}=z(z-\text{1})[(\text{1}-t)C\text{5}-(\text{1}+t)R\text{5}+(R\text{6}-C\text{6})+(xF-L\text{3})(y-\text{1})]$$

The first-order derivatives of z and the set $G\left(x^{*}\right)$ are:


$$F(z)=\frac{dF(z)}{dz}=(\text{2}z-\text{1})[(\text{1}-t)C\text{5}-(\text{1}+t)R\text{5}+(R\text{6}-C\text{6})+(xF-L\text{3})(y-\text{1})]$$

$$G\left(x^{*}\right)=(\text{1}-t)C\text{5}-(\text{1}+t)R\text{5}+(R\text{6}-C\text{6})+(xF-L\text{3})(y-\text{1})$$

**Fig 3:**
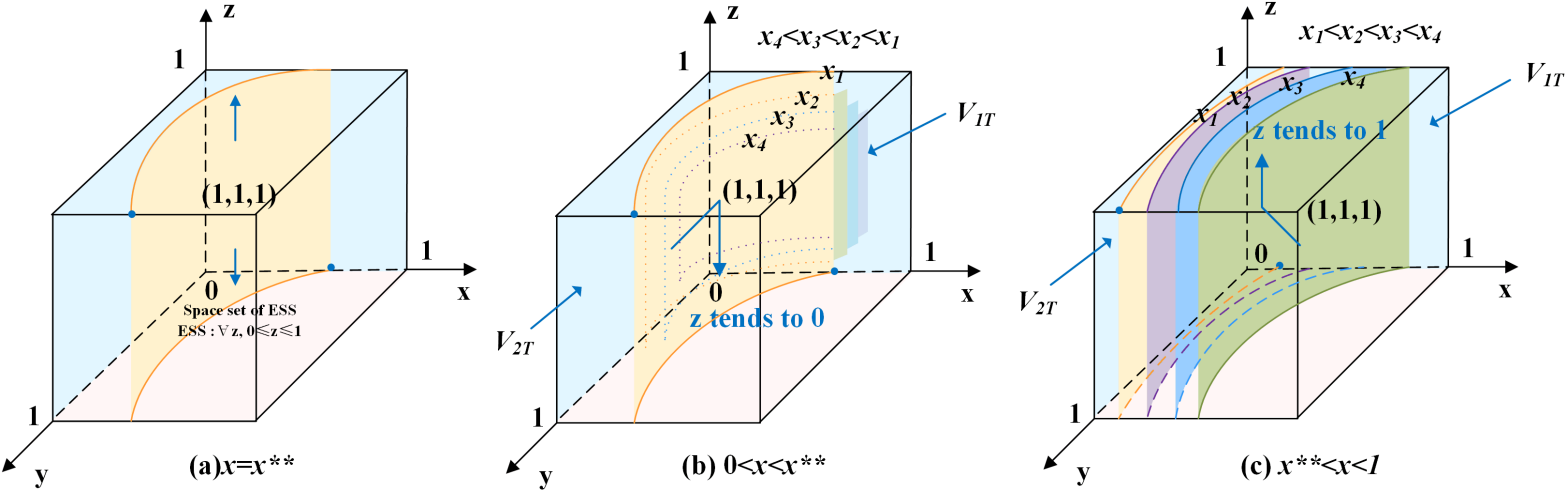
Phase diagram of older adults’ strategy.

According to the stability theorem of differential equations, the following conditions must be satisfied for older adults’ choice of labor participation to be in an equilibrium stable state: F(z)=0 and $\frac{dF(z)}{dz}<\text{0}$. Since $\frac{\partial G(x*)}{\partial x*}<\text{0}$, $G\left(x^{*}\right)$ is a decreasing function with respect to $x^{*}$. Thus, when $x=\frac{(\text{1}-t)C\text{5}-(\text{1}+t)R\text{5}+(R\text{6}-C\text{6})-(y-\text{1})L\text{3}}{(\text{1}-y)F}=x^{**}$, $G(x^{*})=\text{0}$, at which point $\frac{dF(z)}{dz}\equiv \text{0}$, the older adults cannot determine a stabilization strategy. When $x<x^{**}$, $G(x^{*})>\text{0}$, then z=0 is the evolutionary stabilization strategy for older adults. On the contrary, when $x>x^{**}$, $G\left(x^{*}\right)<\text{0}$, then z=1 is the evolutionary stabilization strategy for older adults. The related phase diagram is shown in Fig 3.

Fig 3 shows the volume $V_{\text{2}T}$ of the probability that older adults choose labor participation, and the volume $V_{\text{1}T}$ of the probability that older adults choose non labor participation. When $x<x^{**}$, as shown in Fig 3(b) where $x_{\text{4}}<x_{\text{3}}<x_{\text{2}}<x_{\text{1}}$, the volume of $V_{\text{1}T}$ gradually increases, the volume of $V_{\text{2}T}$ gradually decreases, z gradually approaches 0, and the probability of older adults choosing non-labor participation increases. When $x>x^{**}$, as shown in Fig 3(c) where $x_{\text{1}}<x_{\text{2}}<x_{\text{3}}<x_{\text{4}}$, the volume of $V_{\text{1}T}$ gradually decreases, the volume of $V_{\text{2}T}$ gradually increases, z gradually approaches 1, and the probability of older adults choosing labor participation increases.

The expressions of $V_{\text{1}T}$ and $V_{\text{2}T}$ can be calculated by the following:


$$V_{\text{1}T}=\int_{\text{0}}^{\text{1}}\int_{\text{0}}^{\frac{(\text{1}-t)C\text{5}-(\text{1}+t)R\text{5}+(R\text{6}-C\text{6})+L\text{3}}{L\text{3}}}\frac{(\text{1}-t)C\text{5}-(\text{1}+t)R\text{5}+(R\text{6}-C\text{6})-(y-\text{1})L\text{3}}{(\text{1}-y)F}dydz$$

$$V_{\text{2}T}=\text{1}-V_{\text{1}T}$$

Inference three: The probability of older adults choosing labor participation is positively correlated with the benefit of labor participation, the penalty of negatively hiring older adults for local enterprises, and the gerontological digital inclusion coefficient, and negatively correlated with the employment costs, benefit of non labor participation, and age discrimination.

Proof: Since $\frac{\partial V_{\text{2}T}}{\partial R\text{5}}>\text{0}$, $\frac{\partial V_{\text{2}T}}{\partial C\text{6}}>\text{0}$, $\frac{\partial V_{\text{2}T}}{\partial t}>\text{0}$, $\frac{\partial V_{\text{2}T}}{\partial F}>\text{0}$, $\frac{\partial V_{\text{2}T}}{\partial C\text{5}}<\text{0}$, $\frac{\partial V_{\text{2}T}}{\partial R\text{6}}<\text{0}$, $\frac{\partial V_{\text{2}T}}{\partial L\text{3}}<\text{0}$, when R5, C6, t and F increase, the volume of $V_{\text{2}T}$ grows, and the probability of older adults choosing labor participation increases at this time. When C5, R6 and L3 increase, the volume of $V_{\text{1}T}$ grows, and the probability of older adults choosing non labor participation increases.

Inference three suggests that the higher the gerontological digital inclusion coefficient, and the easier it is for older adults to use digital technology to reduce information barriers and find suitable employment, the higher the probability of choosing labor participation. There is a substitution between the benefits of labor participation and the utility of leisure and the value of household care; the more material income and self-efficacy gains from labor participation, the higher the probability that older adults will choose to participate in the labor market. The stricter the government’s punishment of age discrimination in enterprises, the easier it will be to create an age-friendly employment environment, and thus the higher the probability of obtaining employment security for older adults to choose labor participation. The more serious the age discrimination, the greater the negative impact on the mental health of the older workers, and the lower the probability of the older adults choosing to be employed.

## 5. Stability analysis of the equilibrium point

Setting F(x)=0, F(y)=0 and F(z)=0, the equilibrium points of the system can be derived. For pure strategy equilibrium points, there are $E_{\text{1}}(\text{0},\text{0},\text{0})$, $E_{\text{2}}(\text{0},\text{0},\text{1})$, $E_{\text{3}}(\text{0},\text{1},\text{0})$, $E_{\text{4}}(\text{0},\text{1},\text{1})$, $E_{\text{5}}(\text{1},\text{0},\text{0})$, $E_{\text{6}}(\text{1},\text{0},\text{1})$, $E_{\text{7}}(\text{1},\text{1},\text{0})$ and $E_{\text{8}}(\text{1},\text{1},\text{1})$. For mixed strategy equilibrium points, there are solutions such as $E_{\text{9}}(\text{1},\text{1}+((\text{1}+t)R\text{5}-(\text{1}-t)C\text{5}-(R\text{6}-C\text{6}))/(F\;-\;L\text{3}),(s(R\text{3}+C\text{4})+F+\;L\text{2})/((\text{1}-s)(C\text{3}-C\text{4})+\;L\text{2}\;-\;U\text{2}))$, $E_{\text{1}\text{0}}(-((\text{1}+t)R\text{5}-(\text{1}-t)C\text{5}-(R\text{6}-C\text{6})-L\text{3})/F,\text{0},(rR\text{1}-(\text{1}-r)C\text{1}+C\text{2}+F)/(F-U\text{1}))$, $E_{\text{1}\text{1}}(\text{0},\text{1}-((\text{1}+t)R\text{5}-(\text{1}-t)C\text{5}-(R\text{6}-C\text{6}))/L\text{3},s(R\text{3}+C\text{4})/((\text{1}-s)(C\text{3}-C\text{4})-\;U\text{2}))$, $E_{\text{1}\text{2}}\left(-s(R\text{3}+C\text{4})/(F\;+\;L\text{2}),\text{1}+(rR\text{1}-(\text{1}-r)C\text{1}+C\text{2})/F,\text{0}\right)$, $E_{\text{1}\text{3}}(x^{*},y^{*},z^{*})$and $E_{\text{1}\text{4}}(x^{**},y^{**},z^{**})$. Since x,y,z ∈[0,1], $E_{\text{9}}-E_{\text{14}}$ are meaningful under certain conditions; whereas $E_{\text{12}}$ is meaningless because −s(R3+C4)<0. In order to verify the stability of the equilibrium point, the Jacobian matrix of the three-party evolutionary game system is calculated as follows:


$$\begin{array}{c} J=[\begin{array}{ccc} \frac{\partial F(x)}{\partial x} & \frac{\partial F(x)}{\partial y} & \frac{\partial F(x)}{\partial z}\\ \frac{\partial F(y)}{\partial x} & \frac{\partial F(y)}{\partial y} & \frac{\partial F(y)}{\partial z}\\ \frac{\partial F(z)}{\partial x} & \frac{\partial F(z)}{\partial y} & \frac{\partial F(z)}{\partial z} \end{array}]= \end{array}$$

$$\begin{array}{c} \left[\begin{array}{ccc} (\text{1}\;-\;\text{2}x)\left(\begin{array}{c} rR\text{1}\;-(\text{1}-r)C\text{1}\\ +C\text{2}+(yz-y-z+\text{1})F\\ +zU\text{1} \end{array}\right) & x(\text{1}\;-\;x)(z-\text{1})F & x(\text{1}-x)\left((y-\text{1})F+U\text{1}\right)\\ y(\text{1}-y)(F+(\text{1}-z)L\text{2}) & (\text{1}\;-\;\text{2}y)\left(\begin{array}{c} z(s-\text{1})(C\text{3}-C\text{4})+\\ s(R\text{3}+C\text{4})\\ +xL\text{2}(\text{1}-z)\\ +xF+zU\text{2} \end{array}\right) & y\;(\text{1}-y)\left(\begin{array}{c} \left(s-\text{1})(C\text{3}-C\text{4}\right)\\ +U\text{2}-xL\text{2} \end{array}\right)\\ z(z\;-\;\text{1})(y\;-\;\text{1})\;F & z(z-\text{1})(xF-L\text{3}) & (\text{1}-\text{2}z\;)\left(\begin{array}{c} (\text{1}+t)R\text{5}\\ -(\text{1}-t)C\text{5}-\\ (R\text{6}-C\text{6})-\\ \left(xF-L\text{3})(y-\text{1}\right) \end{array}\right) \end{array}\right] \end{array}$$

Since evolutionarily stable strategies only exist in pure strategies [50], the subsequent discussion will focus on pure strategy points and exclude mixed strategy points from analysis. The Lyapunov first method [51] could be used to judge the stability of the strategy combination. If all eigenvalues of the Jacobi matrix have negative real part, the system equilibrium point would be asymptotically stable, and the system equilibrium point would be stable fixed point of the replicator dynamics. If at least one of the eigenvalues of the Jacobian matrix has a positive real part, the equilibrium point is unstable; If one of the eigenvalues of the Jacobian matrix has a real part of zero, the rest of the eigenvalues have negative real part, the equilibrium point is in a critical state, when the stability can not be determined by the sign of the eigenvalues. Due to space limitations, the equation rR1−(1−r)C1+C2 is simplified as A, the equation (1+t)R5−(1−t)C5−(R6−C6) is simplified as B, and the equation sR3−(1−s)C3+C4+U2 is simplified as C, and the eigenvalues of each system equilibrium point can be obtained as shown in the Table 3:

**Table 3:** Equilibrium point stability analysis.

Type of equilibriums	Balancing point	Jacobian matrix eigenvalues	Symbols	Stability	Conditions
Pure strategy	$E_{\text{1}}(\text{0},\text{0},\text{0})$	λ1=A+F,λ2=s(C4+R3),λ3=B−L3	(×, + ,×)	Unstable	/
	$E_{\text{2}}(\text{0},\text{0},\text{1})$	λ1=−A−F,λ2=s(C4+R3)+F+L2,λ3=B+F−L3	(×, + ,×)	Unstable	/
	$E_{\text{3}}(\text{0},\text{1},\text{0})$	λ1=A,λ2=−s(C4+R3),λ3=B	(×,-,+)	Unstable	/
	$E_{\text{4}}(\text{0},\text{1},\text{1})$	λ1=−A,λ2=−s(R3+C4)−F−L2,λ3=B	(-,-,-)	Stable	(1)
	$E_{\text{5}}(\text{1},\text{0},\text{0})$	λ1=−A,λ2=−s(R3+C4)−F−L2,λ3=B	(×,-,+)	Unstable	/
	$E_{\text{6}}(\text{1},\text{0},\text{1})$	λ1=−A−U1,λ2=C+F,λ3=L3−B−F	(-,-,-)	Stable	(2)
	$E_{\text{7}}(\text{1},\text{1},\text{0})$	λ1=A+U1,λ2=−C,λ3=−B	(-,-,-)	Stable	(3)
	$E_{\text{8}}(\text{1},\text{1},\text{1})$	λ1=−A−U1,λ2=−C,λ3=−B	(-,-,-)	Stable	(4)
Mixed strategy	$E_{\text{9}}(\text{1},y\text{1},z\text{1})$	$\lambda \text{1}=\sqrt{B(B+F-L\text{3})(C+F)}i,\lambda \text{2}=a\text{1},\lambda \text{3}=-\lambda \text{1}$	(0,-,0)	Unsure	/
	$E_{\text{1}\text{0}}(x\text{1},\text{0},z\text{2})$	$\lambda \text{1}=\sqrt{\left(A+F)(A+U\text{1})(B-L\text{3})(\text{1}+F\right)}/F(F-U\text{1})i,\lambda \text{2}=b\text{1},\lambda \text{3}=-\lambda \text{1}$	(0,-,0)	Unsure	/
	$E_{\text{1}\text{1}}(\text{0},y\text{2},z\text{3})$	λ1=A+F(1−y2)(1−z3)+z3U1,λ2=c1,λ3=c2	(+, × ,×)	Unstable	/
	$E_{\text{1}\text{3}}(x^{*},y^{*},z^{*})$	Saddle point			
	$E_{\text{1}\text{4}}(x^{**},y^{**},z^{**})$	Saddle point			

*Notes*: × indicates that the symbol is uncertain and i refers to an imaginary number. (1) rR1+U1−((1−r)C1−C2)<0, sR3+U2−((1−s)C3−C4)<0, L3−(1+t)R5+(1−t)C5+(R6−C6)<0. (2) rR1+U1−((1−r)C1−C2)>0, sR3+U2−((1−s)C3−(C4+F))<0, L3−(1+t)R5+(1−t)C5+(R6−C6)−F<0. (3) rR1+U1−((1−r)C1−C2)<0, sR3+U2−((1−s)C3−C4)>0. (4) rR1+U1−((1−r)C1−C2)<0, sR3+U2−((1−s)C3−(C4+F))>0.

Combined with the previous assumptions, it can be seen that (1+t)R5−(1−t)C5−(R6−C6)>0 and C4+R3>0. The eigenvalues of $E_{\text{1}},\;E_{\text{2}},\;E_{\text{3}}$ and $E_{\text{5}}$ are positive, thus $E_{\text{1}},\;E_{\text{2}},\;E_{\text{3}}$ and $E_{\text{5}}$ are unstable points.

Inference four: When rR1+U1−((1−r)C1−C2)<0, sR3+U2−((1−s)C3−C4)<0 and  L3−(1+t)R5+(1−t)C5+(R6−C6)<0, condition (1) is satisfied, and $E_{\text{4}}(\text{0},\text{1},\text{1})$ is the stable fixed point of the replicator dynamics. When rR1+U1−((1−r)C1−C2)<0 and sR3+U2−((1−s)C3−C4)>0, condition (3) is satisfied and $E_{\text{7}}(\text{1},\text{1},\text{0})$ is the stable fixed point of the replicator dynamics.

Inference four suggests that when rR1+U1−((1−r)C1−C2)<0 is true, the additional social welfare that the government department receives when it acts positively and the gains from digital technology are not enough to offset the extra cost of choosing to act positively, and the government department then tends to choose the strategy of negative action. When sR3+U2−((1−s)C3−C4)<0 is true, it indicates that the additional gain obtained by local enterprises choosing to positively employ older adults as well as the gain from the digital transformation of the enterprise is smaller than the difference between the cost of older workers labor and the cost of alternative labor, and then the local enterprises tend to choose to negatively employ older adults. When L3−(1+t)R5+(1−t)C5+(R6−C6)<0, it indicates that although subject to some age discrimination, the net benefit of older adults choosing to participate in the labor force is greater than the net benefit of choosing not to participate in the labor force, at which time older adults tend to choose to delay retirement or re-employment.

Inference five: When rR1+U1−((1−r)C1−C2)>0, sR3+U2−((1−s)C3−(C4+F))<0 and  L3−(1+t)R5+(1−t)C5+(R6−C6)−F<0, condition (2) is satisfied, and $E_{\text{6}}(\text{1},\text{0},\text{1})$ is the stable fixed point of the replicator dynamics. When rR1+U1−((1−r)C1−C2)>0 and sR3+U2−((1−s)C3−(C4+F))>0, condition (4) is satisfied and $E_{\text{8}}(\text{1},\text{1},\text{1})$ is the stable fixed point of the replicator dynamics.

Inference five shows that when rR1+U1−((1−r)C1−C2)>0, the construction of digital government plays a crucial role in reducing the operating cost of government departments; at this time, the additional social welfare that the government department receives when it acts positively and the gains from digital technology are great enough to offset the extra cost of positive action, and therefore the government department tends to choose the strategy of positive action. When sR3+U2−((1−s)C3−(C4+F))>0, the additional gain obtained by local enterprises choosing to positively employ older adults as well as the gain from the digital transformation of the enterprise are large enough to offset the difference in employment costs as well as the impact of government fines, and thus local enterprise tends to positively employ older adults at this point. When L3−(1+t)R5+(1−t)C5+(R6−C6)−F<0, it indicates that the net benefit of older adults choosing to participate in the labor force and the gain or loss due to age discrimination under the government’s positive action are greater than the net benefit of choosing not to participate in the labor force, and then older adults still tend to choose to delay their retirement or re-employment. These findings are further corroborated by the stochastic analysis in S1 File, which shows that the stable fixed point remains robust under stochastic noise.

## 6. Simulation analysis

### 6.1. Evolutionary stable strategy analysis

To verify the validity of the evolutionary stability analysis, the initial parameter values are set with reference to the research of Dong et al. [39]. For the parameters not covered therein, assignments are made in accordance with the conditions of the stable fixed points. MATLAB 2016a is used for numerical simulation. The initial values of parameters are set as follows: C1=3,C2=2,R3=3,C3=2.5,C4=1,R5=3,C5=1,R6=2,C6=1.5,F=0.5,L1=1.5,U2=0.2,L2=1.5,L3=1,t=0.2.

**Fig 4:**
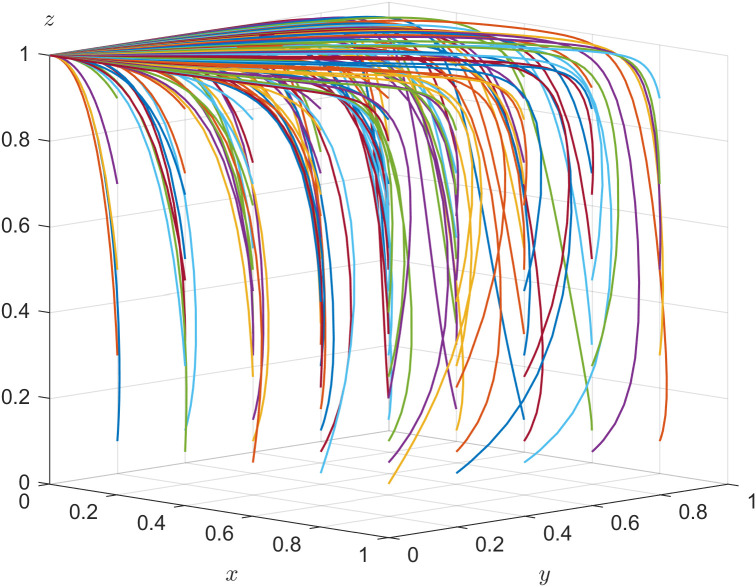
Evolutionary stability of ${𝐄}_{4}$.

In Inference four, r=0.1,R1=−0.5  and U1=0.2 are set to satisfy rR1+U1−((1−r)C1−C2)<0, and s=0.1 is set to satisfy sR3+U2−((1−s)C3−C4)<0. The validity test of the system evolution stability of $E_{\text{4}}$ is shown in Fig 4. In Fig 4, all the points finally converge to $E_{\text{4}}(\text{0},\text{1},\text{1})$ after evolving 50 times, which verifies the conclusion that $E_{\text{4}}(\text{0},\text{1},\text{1})$ is a stable point in Inference four. Regardless of the initial strategies of the three parties, $E_{\text{4}}(\text{0},\text{1},\text{1})$ is the system’s evolutionary stable strategy when the constrained conditions are fulfilled, and the strategic choice for government department, local enterprise and older adults is {negative action, positively hiring older adults, labor participation}.

**Fig 5:**
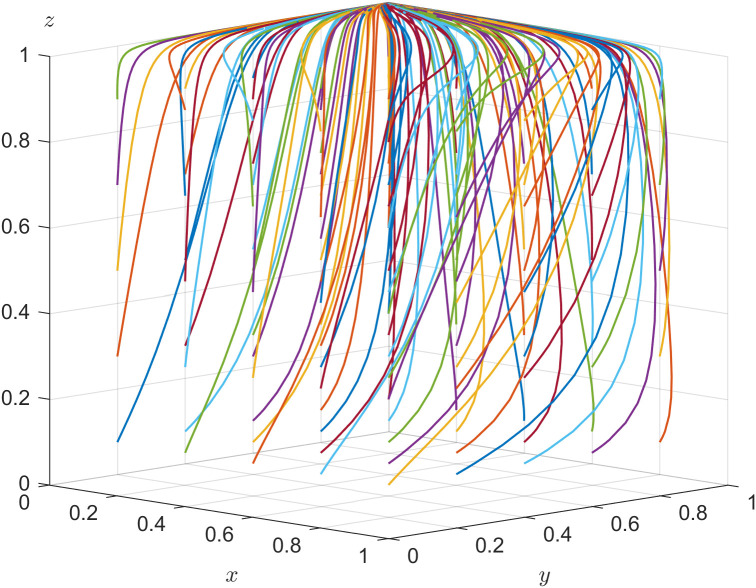
Evolutionary stability of ${𝐄}_{7}$.

The enterprise digital transformation efficacy coefficient is adjusted and s=0.3 is set to satisfy sR3+U2−((1−s)C3−C4)>0. Other parameters are assigned unchanged. The validity test of the system evolution stability of $E_{\text{7}}$ is shown in Fig 5. The different colored lines in Fig 5 illustrate the tripartite evolutionary trajectory with nonfixed initial strategies after evolving 50 times, which converge to the equilibrium point $E_{\text{7}}(\text{1},\text{1},\text{0})$, indicating that the ESS for the government department, local enterprise, and older adults is {positive action, positively hiring older adults, non-labor participation} when the constrained conditions are satisfied.

**Fig 6:**
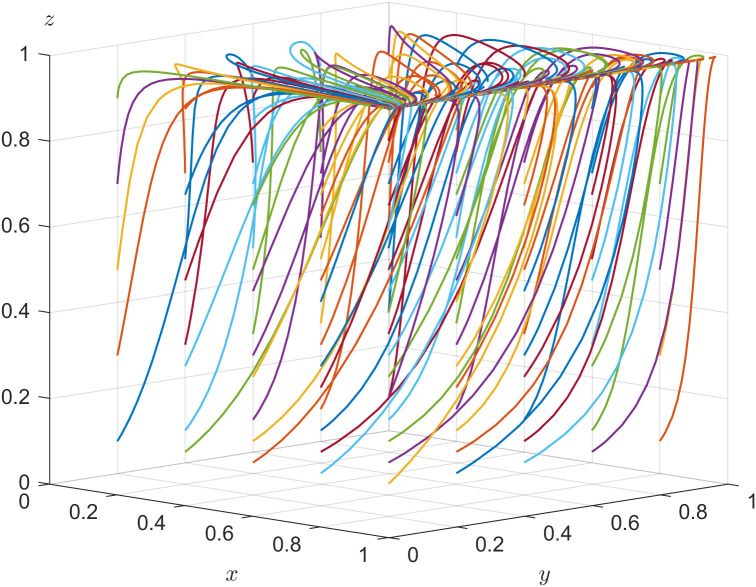
Evolutionary stability of ${𝐄}_{6}$.

In Inference five, r=0.3,R1=1  and U1=0.5 are set to satisfy rR1+U1−((1−r)C1−C2)>0, and s=0.1 is set to satisfy sR3+U2−((1−s)C3−(C4+F))<0. The validity test of the system evolution stability of $E_{\text{6}}$ is shown in Fig 6. After evolving 50 times, all the points finally converge to $E_{\text{6}}(\text{1},\text{0},\text{1})$, which verifies the conclusion that $E_{\text{6}}(\text{1},\text{0},\text{1})$ is a stable point in Inference five. Regardless of the initial strategies of the three parties, $E_{\text{6}}(\text{1},\text{0},\text{1})$ is the system’s evolutionary stable strategy when the constrained conditions are fulfilled. As shown in Fig 6, the corresponding strategic choice for government department, local enterprise and older adults is {positive action, negatively hiring older adults, labor participation}.

**Fig 7:**
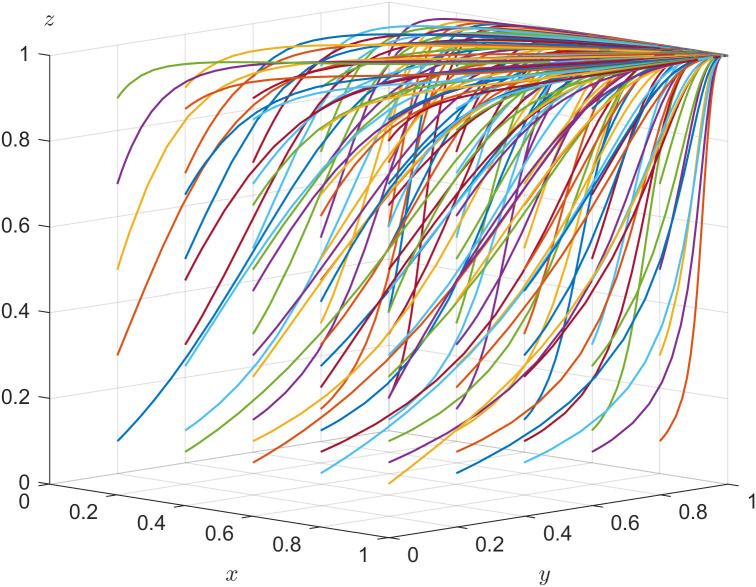
Evolutionary stability of ${𝐄}_{8}$.

The enterprise digital transformation efficacy coefficient is adjusted and s=0.3 is set to satisfy sR3+U2−((1−s)C3−(C4+F))>0. Other parameters are assigned unchanged. The validity test of the system evolution stability of $E_{\text{8}}$ is shown in Fig 7. The different colored lines in Fig 7 illustrate the tripartite evolutionary trajectory with nonfixed initial strategies after evolving 50 times, which converge to the equilibrium point $E_{\text{8}}(\text{1},\text{1},\text{1})$. The corresponding strategic choice for the government department, local enterprise, and older adults is {positive action, positively hiring older adults, labor participation} when the constrained conditions are satisfied.

### 6.2. Government department sensitivity analysis

**Fig 8:**
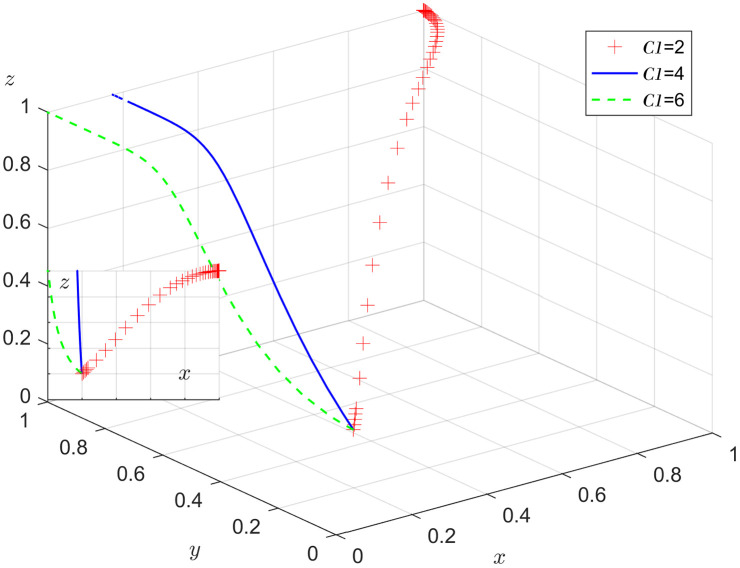
Impact of the cost of positive action.

Under the condition that other parameters remain unchanged, the results of replicating the system of dynamic equations evolving 50 times over time by assigning C1=2, 4, 6 respectively, are shown in Fig 8. Fig 8 shows that as the system evolves to the point of stability, the cost of positive action reduces the probability of government department choosing positive action. As C1 exceeds a certain threshold, x tends to converge to 0 and the probability of the government department choosing negative action rises.

**Fig 9:**
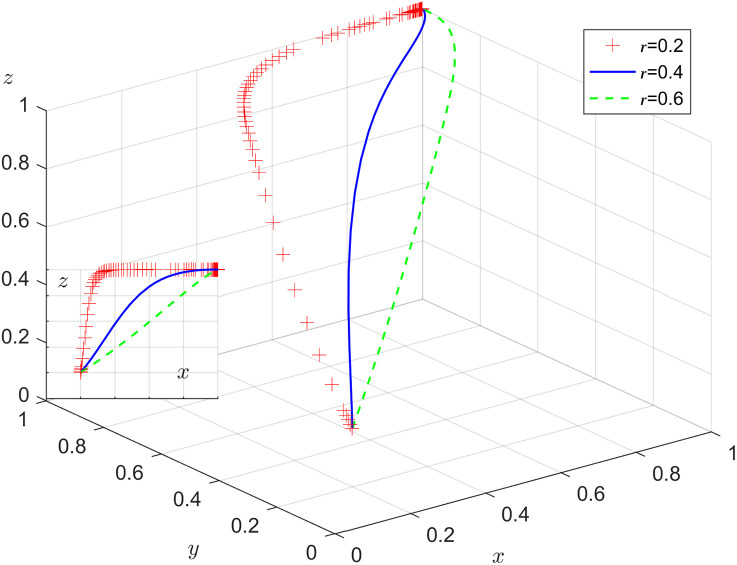
Impact of government digitalization efficacy coefficient.

Under the condition that other parameters remain unchanged, the results of replicating the system of dynamic equations evolving 50 times over time by assigning r=0.2, 0.4, 0.6 respectively, are shown in Fig 9. Fig 9 shows that in the process of system evolution to a stable point, the increase in the government digitalization efficacy coefficient can accelerate the evolution speed of the government’s stable choice of positive action. With the increase of r, the faster x tends to 1, the probability of the government department choosing positive action, i.e., the construction of the digital government can effectively improve the efficiency of the government’s public services, which prompts the government department to choose positive action.

### 6.3. Local enterprise sensitivity analysis

**Fig 10:**
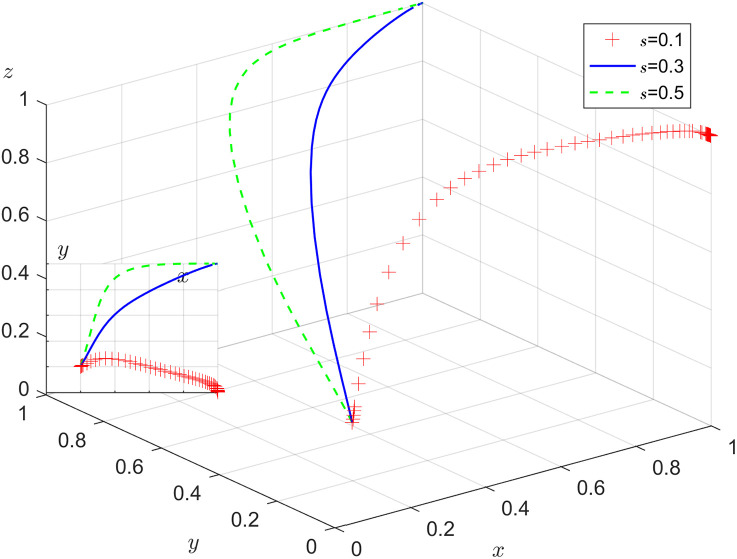
Impact of enterprise digital transformation efficacy coefficient.

Under the condition that other parameters remain unchanged, the results of replicating the system of dynamic equations evolving 50 times over time by assigning s=0.1, 0.3, 0.5 respectively, are shown in Fig 10. Fig 10 shows that in the process of system evolution to the stabilization point, there is a certain threshold effect on the positive impact of enterprise digital transformation on its active employment of older adults. When less than the threshold value, the change in the enterprise digital transformation efficacy coefficient is not enough to affect the enterprise to actively employ older adults. When more than the threshold value, the increase in the enterprise digital transformation efficacy coefficient can accelerate the evolution of the enterprise’s stable choice to positive action. With the increase in s, the faster y tends to 1, the probability of the enterprise’s choice of positively employing older adults rises.

**Fig 11:**
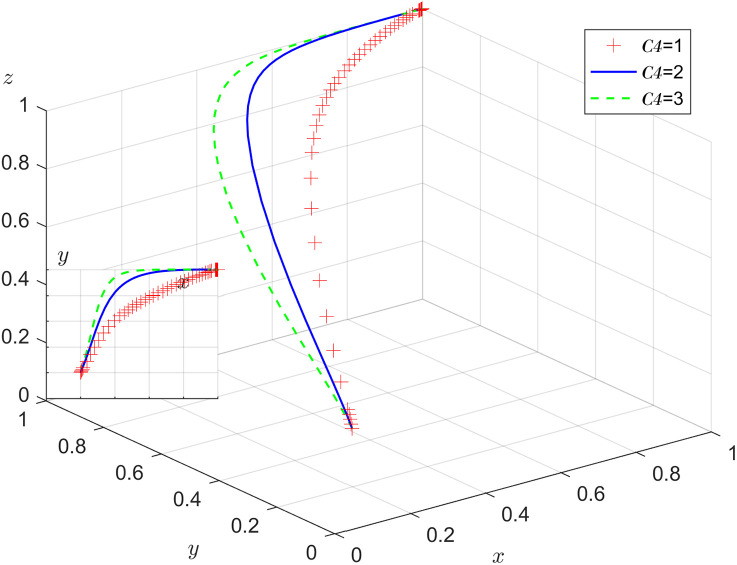
Impact of the cost of alternative labor.

Under the condition that other parameters remain unchanged, the results of replicating the system of dynamic equations evolving 50 times over time by assigning C4=1, 2, 3 respectively, are shown in Fig 11. Fig 11 shows that as the system evolves to the stabilization point, an increase in the cost of alternative labor will increase the probability of the enterprise positively employing older adults. As C4 increases, the faster y converges to 1, the higher the probability of the enterprise positively employing older adults.

### 6.4. Older adults sensitivity analysis

**Fig 12:**
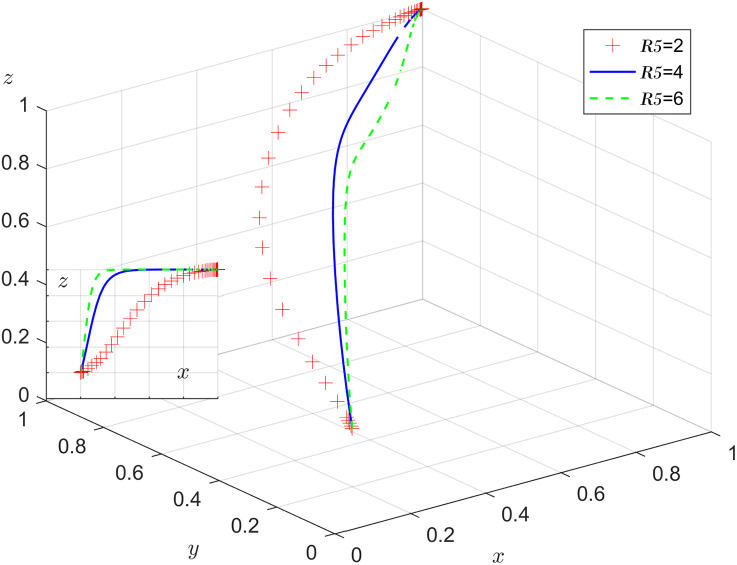
Impact of the benefit of labor participation.

Under the condition that other parameters remain unchanged, the results of replicating the system of dynamic equations evolving 50 times over time by assigning R5=2, 4, 6 respectively, are shown in Fig 12. As the system evolves to a stable point, increases in employment income and self-efficacy can accelerate the evolution of stable choice of labor participation by older adults. As R5 increases, the faster z converges to 1, the higher the probability of older adults choosing labor participation.

**Fig 13:**
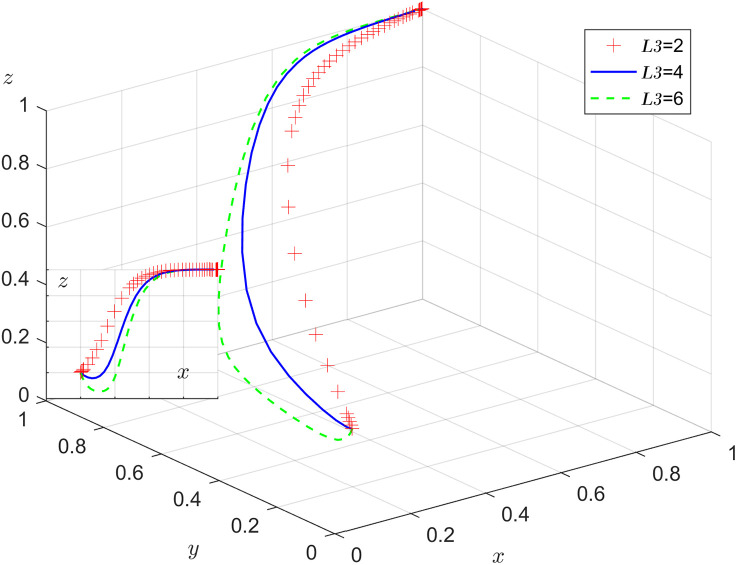
Impact of age discrimination.

Under the condition that other parameters remain unchanged, the results of replicating the system of dynamic equations evolving 50 times over time by assigning L3=2, 4, 6 respectively, are shown in Fig 13. As the system evolves to a stable point, the negative impact of age discrimination slows down the evolution of the stable choice of labor participation by older adults. As L3 increases, the slower z converges to 1 and the probability of older adults choosing labor participation decreases.

## 7. Discussion and conclusion

### 7.1. Conclusion

This study developed a tripartite evolutionary game model involving government departments, local enterprises, and older adults to analyze how digital transformation and age-related employment conditions jointly influence strategic choice and their evolutionary dynamics. Rather than focusing on country-specific parameter value or policy instruments, the conclusions are framed at the level of strategic mechanisms that govern interactions among these three parties. Numerical simulations implemented in MATLAB demonstrate that the stability and direction of strategy evolution are closely linked to changes in the relative costs and benefits faced by each actor.

For government departments, the magnitude of the government digitalization efficacy coefficient affects government department’s strategic choice. When the size of government digitalization efficacy coefficient is small, the low level of digital government construction cannot offset the extra cost of positive action. In this situation, the government tends to choose passive action. Conversely, high digitalization efficacy encourages active involvement due to greater net benefits.

For local enterprises, both the digital transformation efficacy coefficient and labor costs shape enterprise decisions. When the enterprise digital transformation efficacy coefficient is small, the additional benefits from positively employing older adults and digital technology cannot offset the additional costs of positively employing older adults, and enterprises tend to choose to negatively employ older adults. However, when digital tools and age-inclusive investments reduce costs and enhance productivity, enterprises are more likely to employ older workers.

For older adults, employment income, self-efficacy, and perceived age discrimination are key determinants of older adults’ behaviors. When employment income and self-efficacy enhancement are greater, older adults receive greater benefits from labor participation and are more likely to choose to delay retirement or re-employment. In contrast, stronger age discrimination diminishes their labor participation.

These conclusions are further validated by the stochastic evolutionary game extension in S1 File, which demonstrates that the strategic mechanisms identified in the deterministic analysis remain robust under stochastic noise.

### 7.2 Policy implications

Enhancing labor participation of older adults requires the coordination of the interests of government departments, local enterprises, and older workers. The findings point to several areas where policy interventions could reduce constraints, though the specific instruments will vary across institutional contexts.

Firstly, the digital governance framework should be strengthened by promoting the application of government data in employment services. Strategic investments in digital infrastructure must prioritize the development of government cloud platforms and data sharing systems. Special attention should be given to creating age-friendly employment service platforms that provide targeted assistance to silver-haired individuals possessing both labor capacity and employment intentions. Secondly, enterprise digital transformation should be empowered to construct age-inclusive digital workplaces. A dedicated fiscal support mechanism should be established to offer digital transformation subsidies, incentivizing enterprises to upgrade age-adaptive production facilities, thereby reducing employment barriers for older workers. Differentiated social security policies could be implemented, including social security contribution reductions or income tax deductions for enterprises that employ older workers above specified thresholds. Government special funds should compensate enterprises appropriately to alleviate the aging workforce costs. Thirdly, whether older adults choose to stay in or return to work depends on both financial returns and perceived employability. Policies that recognize their experience, certify their skills, or otherwise validate their workplace value can work on both fronts, improving expected earnings while boosting confidence. The specific design of such measures should be adapted to local institutional and cultural contexts, rather than assumed to be universally transferable. Finally, institutional safeguards against age discrimination influence older adults’ expected returns from labor participation. Strengthening enforcement mechanisms and increasing the expected costs of discriminatory practices, such as tougher penalties, better monitoring, or mandatory public reporting, raises the cost of discriminatory behavior and can shift how employers act. The specific tools will depend on what legal systems and enforcement mechanisms are already in place, but the basic principle holds across contexts.

Overall, these policy implications underscore that shifting the system away from low-efficiency outcomes is unlikely to be achieved through isolated interventions. Coordinated measures that simultaneously improve digital governance, enterprise capabilities, and older adults’ employment conditions are more likely to facilitate convergence toward higher-welfare equilibria. While these examples draw on the Chinese institutional context, the underlying logic of reducing binding constraints and enabling coordination across actors may be relevant for other aging societies navigating digital transformation. The specific policy tools will differ, but the need for multi-actor coordination likely generalizes.

### 7.3. Discussion

This study examines how government departments, local enterprises, and older adults adjust their strategies when facing population aging and digital transformation. Although China is used as the empirical setting, the purpose of the model is to identify mechanism-level dynamics that are not inherently country-specific, rather than to derive context-bound policy prescriptions. By adopting an evolutionary game approach, the analysis reflects a basic feature of real-world decision-making. Actors typically operate under bounded rationality, adjusting their strategies over time based on observed outcomes rather than instantaneously optimizing under full information. This perspective helps explain why societies facing similar demographic pressures may nonetheless exhibit divergent regulatory practices and employment patterns. In our framework, such cross-country differences could be captured through alternative parameter configurations. These configurations reflect variations in welfare regimes, labor market institutions, and digital development levels, rather than changes in the model’s underlying strategic structure. Conceptually, the key novelty of the model lies not in the mere inclusion of multiple actors, but in revealing how digitalization generates cross-actor interactions that can either jointly unlock high-participation equilibria or trap the system in a low-activity state.

The model has clear limitations. Government departments, local enterprises, and older adults are treated as homogeneous groups, which abstract from substantial heterogeneity in organizational capacity, digital readiness, health status, and work experience. Government departments face heterogeneous budget constraints. Local enterprises vary significantly in size and digital capacity. Older workers vary in education, health, and work history. Capturing this heterogeneity can illustrate how strategies diverge across subgroups and whether targeted interventions outperform blanket approaches. We also assume actors learn by comparing themselves to similar others, but people often learn across groups or form coalitions. Extending the framework to capture these dynamics could enrich the analysis of strategic adjustment.

Another limitation is that, while the model identifies conditions under which different equilibria become stable, it does not explicitly trace the transition paths between equilibria. Knowing whether shifts happen smoothly or through disruptive periods would help design policies meant to move systems toward better outcomes. Relatedly, the set of initial conditions leading to a particular equilibrium and potential path dependence are not explicitly analyzed, though they matter for assessing the feasibility and timing of policy-induced transitions.

Future research could apply the model to different institutional contexts to test the robustness of its core mechanisms through comparative parameter calibration. Second, introducing cohort effects could capture how successive generations of older adults enter the labor market with different digital skills. Third, linking labor market outcomes back to demographic processes, such as how job opportunities affect when people retire, would further deepen understanding of how aging societies adapt to digital transformation over the long run.

## Supporting information

S1 File Supplementary methods, additional analyses, and figures. (DOCX)
