# Supplementary material for: Rejection or support? research on labor participation strategy of older adults based on three-party evolutionary game
Source: PLoS One. 2026 Apr 7;21(4):e0346531. doi: 10.1371/journal.pone.0346531 (PMC13056212; doi:10.1371/journal.pone.0346531)
Supplement: S1 File — (DOCX) [file pone.0346531.s001.docx]

**Supplementary methods, additional analyses, and figures**

**1. Construction of Stochastic Evolutionary Game Model**

Considering the complexity, variability of real-world conditions and the interference of uncertain factors, which may affect the strategy selection of the three parties, it is necessary to add random factors to the evolutionary game model. In this section, Gaussian white noise is introduced into the replication dynamic equations:

$$\begin{aligned} dx\left( t \right)=x\left( t \right)\left( 1-x\left( t \right) \right)\left[ rR1-\left( 1-r \right)C1+C2+zU1+\left( y-1 \right)\left( z-1 \right)F \right]dt+\sigma x\left( t \right)\left( 1-x\left( t \right) \right)d\omega\left( t \right)\#\left( 1 \right) \end{aligned}$$

$$\begin{aligned} dy\left( t \right)=y\left( t \right)\left( 1-y\left( t \right) \right)\left[ z\left( s-1 \right)\left( C3-C4 \right)+s\left( R3+C4 \right)+xF+zU2+x\left( 1-z \right)L2 \right]dt+\sigma y\left( t \right)\left( 1-y\left( t \right) \right)d\omega\left( t \right)\#\left( 2 \right) \end{aligned}$$

$$\begin{aligned} dz\left( t \right)=z\left( t \right)\left( 1-z\left( t \right) \right)\left[ \left( t-1 \right)C5+\left( 1+t \right)R5-\left( R6-C6 \right)+\left( xF-L3 \right)\left( 1-y \right) \right]dt+\sigma z\left( t \right)\left( 1-z\left( t \right) \right)d\omega\left( t \right)\#\left( 3 \right) \end{aligned}$$

In these equations, $\sigma$ represents the intensity of random disturbance and $\omega(t)$ represents one-dimensional standard Brownian motion. The Brownian motion represents a random fluctuation phenomenon, and it can well reflect the influence of random disturbance in the evolution of groups. $d\omega(t)$ is Gaussian white noise, when $t>0$ and time step $h>0$, its increment $\Delta\omega\left( t \right)=\omega\left( t+h \right)-\omega(t)$ follows a normal distribution $N(0,\sqrt{h})$.

**2. Stability Analysis of Evolutionary Equilibrium Solution**

Assuming that the initial moment is $t=0$, the initial values are $x\left( 0 \right)=0$, $y\left( 0 \right)=0$, and $z\left( 0 \right)=0$, and we can find that $x\left( t \right)=0$, $y\left( t \right)=0$, and $z\left( t \right)=0$ are the equilibrium solutions of equations(1)-(3). That is, without external interference, the system will always stay in the state where the government departments choose positive action, the local enterprises choose positively hiring older adults, and older adults choose labor participation. However, due to the high uncertainty in the real world, it is often impossible to achieve this ideal situation, and each group member will always be more or less disturbed by random factors. Thus, it is necessary to consider the influence of random disturbance on the stability of the system. The sufficient criterion for the stability of the SDE(stochastic dynamic equation) is given below, and the stability of strategy selection is judged.

***Theorem 1.***A stochastic differential equation is given as[1]:

$$\begin{aligned} dx\left( t \right)=f\left( t,x\left( t \right) \right)dt+g\left( t,x\left( t \right) \right)d\omega\left( t \right), x\left( t_{0} \right)=x_{0}\#\left( 4 \right) \end{aligned}$$

Let $x\left( t \right)=x(t,x_{0})$be the solution of equation (4), given a continuously differentiable function $V\left( t,x \right)$ and positive constants; there exists $c_{1}\left| x \right|^{p}\leq V\left( t,x \right)\leq c_{2}\left| x \right|^{p}, t\geq0$. Let $LV\left( t,x \right)=V_{t}\left( t,x \right)+V_{x}\left( t,x \right)f\left( t,x \right)+\frac{1}{2}g^{2}(t,x)V_{xx}\left( t,x \right)$:

1) If there is a positive constant $\gamma$, which satisfies $LV\left( t,x \right)\leq-\gamma V\left( t,x \right),t\geq0$, then the zero solution p-th moment of equation (4) is exponentially stable, and $E\left| x\left( t,x_{0} \right) \right|^{p}<\frac{c2}{c1}\left| x_{0} \right|^{p}e^{-\gamma t}, t\geq0$.

2) If there is a positive constant $\gamma$, which satisfies $LV\left( t,x \right)\geq\gamma V\left( t,x \right),t\geq0$, then the zero solution p-th moment of equation (4) is exponentially stable, and $E\left| x\left( t,x_{0} \right) \right|^{p}\geq\frac{c2}{c1}\left| x_{0} \right|^{p}e^{-\gamma t}, t\geq0$.

For equations(1)-(3), let $V\left( t,x \right)=x, V\left( t,y \right)=y, V\left( t,z \right)=z, c1=1, c2=1, p=1$ and $\gamma=1$; then, $LV\left( t,x \right)=f\left( t,x \right), LV\left( t,y \right)=f\left( t,y \right)$ and $LV\left( t,z \right)=f\left( t,z \right).$ If the zero solution p-th moment exponential of equations(1)-(3) is stable, it needs to satisfy

$$\begin{aligned} \left[ rR1-\left( 1-r \right)C1+C2+zU1+\left( y-1 \right)\left( z-1 \right)F \right]x\leq-x\#\left( 5 \right) \end{aligned}$$

$$\begin{aligned} \left[ z\left( s-1 \right)\left( C3-C4 \right)+s\left( R3+C4 \right)+xF+zU2+x\left( 1-z \right)L2 \right]y\leq-y\#\left( 6 \right) \end{aligned}$$

$$\begin{aligned} \left[ \left( t-1 \right)C5+\left( 1+t \right)R5-\left( R6-C6 \right)+\left( xF-L3 \right)\left( 1-y \right) \right]z\leq-z\#\left( 7 \right) \end{aligned}$$

According to $x, y, z\in[0,1]$, equations(5)-(7) are reduced correspondingly, and three sufficient conditions are obtained to satisfy the above equations:

$$\left[ rR1-\left( 1-r \right)C1+C2+zU1+(y-1)(z-1)F \right]\leq-1$$

$$\left[ z\left( s-1 \right)\left( C3-C4 \right)+s\left( R3+C4 \right)+xF+zU2+x(1-z)L2 \right]\leq-1$$

$$\left[ \left( t-1 \right)C5+\left( 1+t \right)R5-(R6-C6)+(xF-L3)(1-y) \right]\leq-1$$

When the above three conditions are satisfied at the same time, the zero solution p-th moment exponential of equations(1)-(3) is stable. This means that, as the time goes on, the proportion of noncooperative strategies (the government departments choose negative action, the local enterprises choose negatively hiring older adults and the older adults choose non-labor participation) will exponentially decay to zero. At this time, the only evolutionarily stable strategy is a cooperative strategy (the government departments choose positive action, the local enterprises choose positively hiring older adults and the older adults choose labor participation).

**3. Numerical Simulation and Analysis**

**3.1 Stochastic Taylor Expansion of Replication Dynamic Equation.**

Since equations(1)-(3) are all nonlinear $It\hat{o}'s$ SDEs, it is impossible to directly obtain the analytical solutions, so the stochastic Taylor expansion is used to solve the equations numerically. We adopt the Milstein numerical method to solve the equations. Therefore, based on the above principles, the corresponding Taylor expansions for the government departments, local enterprises, and the older adults can be derived.

$$x\left( t_{n+1} \right)=x\left( t_{n} \right)+h\left[ rR1-\left( 1-r \right)C1+C2+zU1+\left( y-1 \right)\left( z-1 \right)F \right]x\left( t_{n} \right)+\Delta\omega_{n}\sigma x\left( t_{n} \right)+\frac{1}{2}\left[ {(\Delta\omega_{n})}^{2}-h \right]\sigma^{2}x\left( t_{n} \right)+\frac{1}{2}h^{2}{[rR1-\left( 1-r \right)C1+C2+zU1+(y-1)(z-1)F]}^{2}x\left( t_{n} \right)+R$$

$$y\left( t_{n+1} \right)=y\left( t_{n} \right)+h\left[ z\left( s-1 \right)\left( C3-C4 \right)+s\left( R3+C4 \right)+xF+zU2+x(1-z)L2 \right]y\left( t_{n} \right)+\Delta\omega_{n}\sigma y\left( t_{n} \right)+\frac{1}{2}\left[ {(\Delta\omega_{n})}^{2}-h \right]\sigma^{2}y\left( t_{n} \right)+\frac{1}{2}h^{2}{[z\left( s-1 \right)\left( C3-C4 \right)+s\left( R3+C4 \right)+xF+zU2+x(1-z)L2]}^{2}y\left( t_{n} \right)+R$$

$$z\left( t_{n+1} \right)=z\left( t_{n} \right)+h\left[ \left( t-1 \right)C5+\left( 1+t \right)R5-(R6-C6)+(xF-L3)(1-y) \right]z\left( t_{n} \right)+\Delta\omega_{n}\sigma z\left( t_{n} \right)+\frac{1}{2}\left[ {(\Delta\omega_{n})}^{2}-h \right]\sigma^{2}z\left( t_{n} \right)+\frac{1}{2}h^{2}{[\left( t-1 \right)C5+\left( 1+t \right)R5-(R6-C6)+(xF-L3)(1-y)]}^{2}z\left( t_{n} \right)+R$$

According to these equations, the numerical solution of the $It\hat{o}'s$ SDEs(1)-(3) can be realized, and the corresponding equilibrium solution can be obtained.

**3.2 Parameters Sensitivity Analysis and Discussion.**

To reveal the key elements and potential future development trends in the evolutionary process of highly uncertain events, we use Matlab2016a to conduct numerical simulations on the stochastic evolutionary game model. The values of each parameter were assigned with reference to the manuscript. The initial probabilities were set as $x=y=z=0.2$. The intensity of the random disturbance term is set as $\sigma=0.2, 0.4, 0.6$, respectively, to investigate the impact of stochastic disturbance intensity on the evolutionary trends. The simulation results are shown in S1 Fig, S2 Fig and S3 Fig. In the presence of random disturbances, the evolutionary strategic behaviors of the three game participants exhibit a certain degree of fluctuations, but ultimately converge to the strategy of {positive action, positively hiring older adults, labor participation}. As the disturbance intensity increases, the participants become more sensitive to changes in stochastic interference, undergoing more intense fluctuations. Under different disturbance intensities, the speed at which the three game participants evolve to a stable strategy varies.


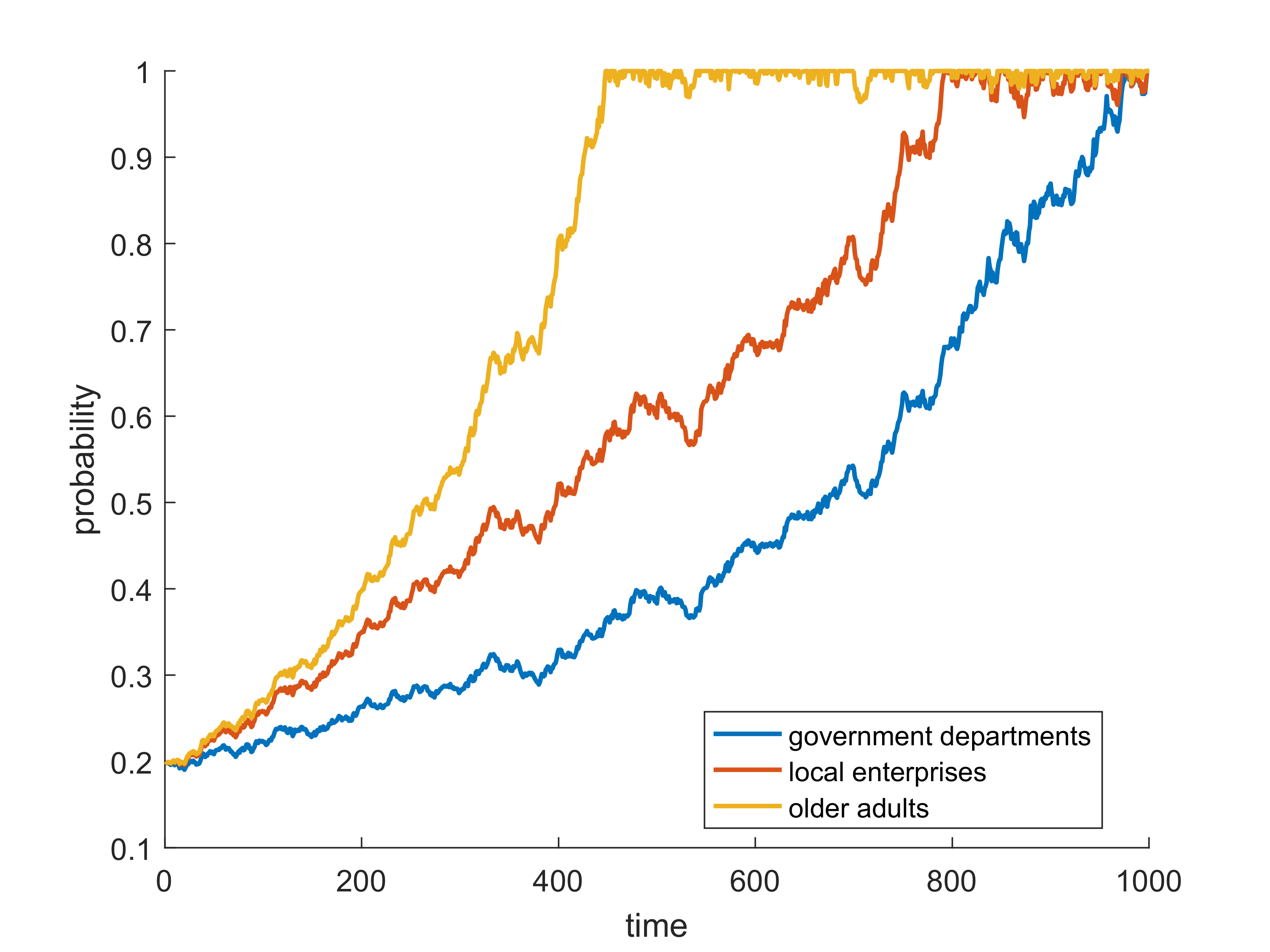


**S1 Fig.** $\boldsymbol{\sigma}\mathbf{=0.2}$


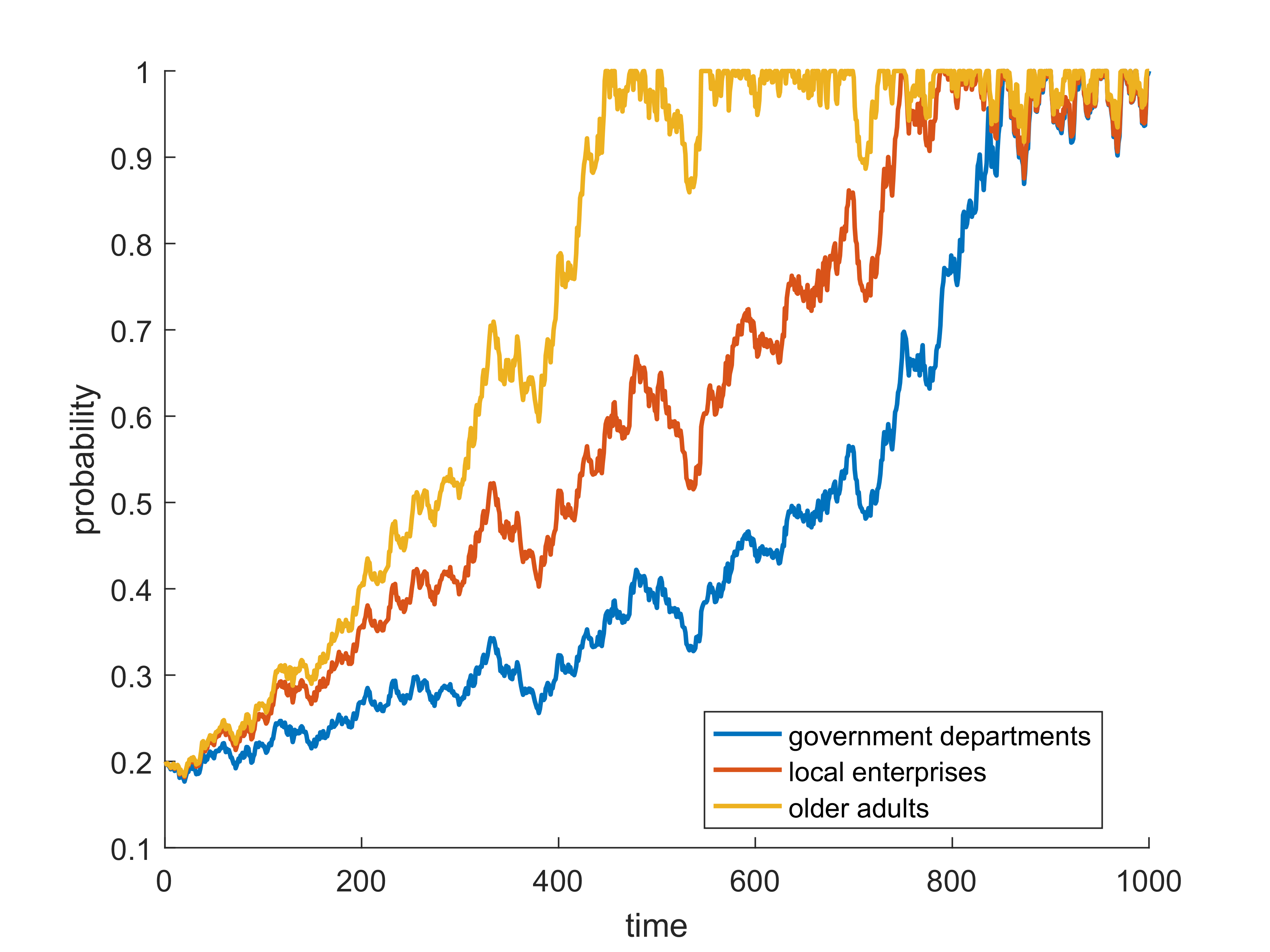


**S2 Fig.** $\boldsymbol{\sigma}\mathbf{=0.4}$


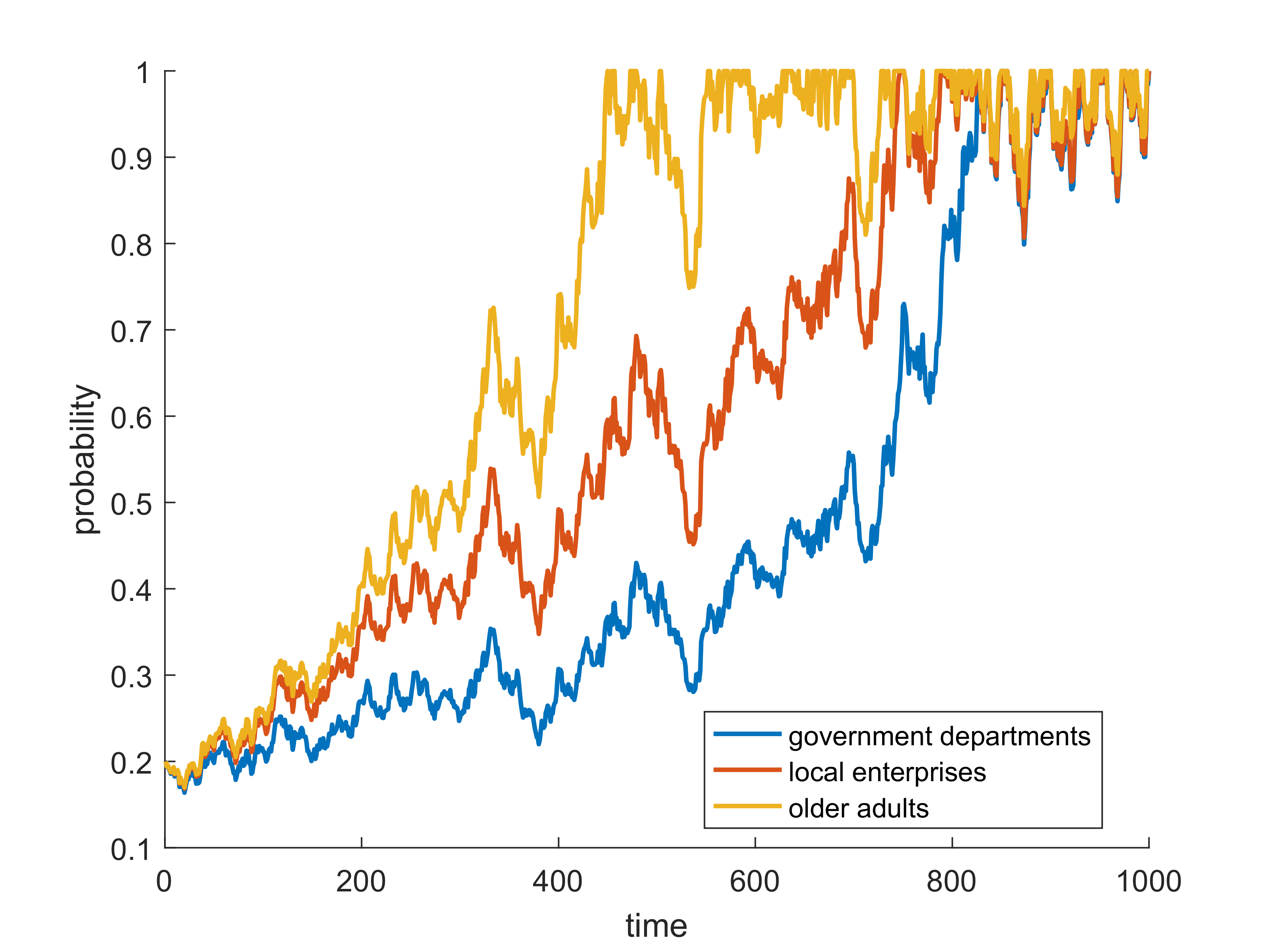


**S3 Fig.** $\boldsymbol{\sigma}\mathbf{=0.6}$

With other parameters remaining unchanged, we assume $r=0.1, 0.3, 0.5$. S4 Fig illustrates the evolutionary trend of government departments’ strategies under different intensities of government digitalization efficacy. When $r$ is small, government departments tend to choose negative action; when $r$ is large, they tend to adopt positive actions. As $r$ increases, the speed at which $x$ tends to 1 becomes faster, and the probability of government departments choosing positive actions rises accordingly.


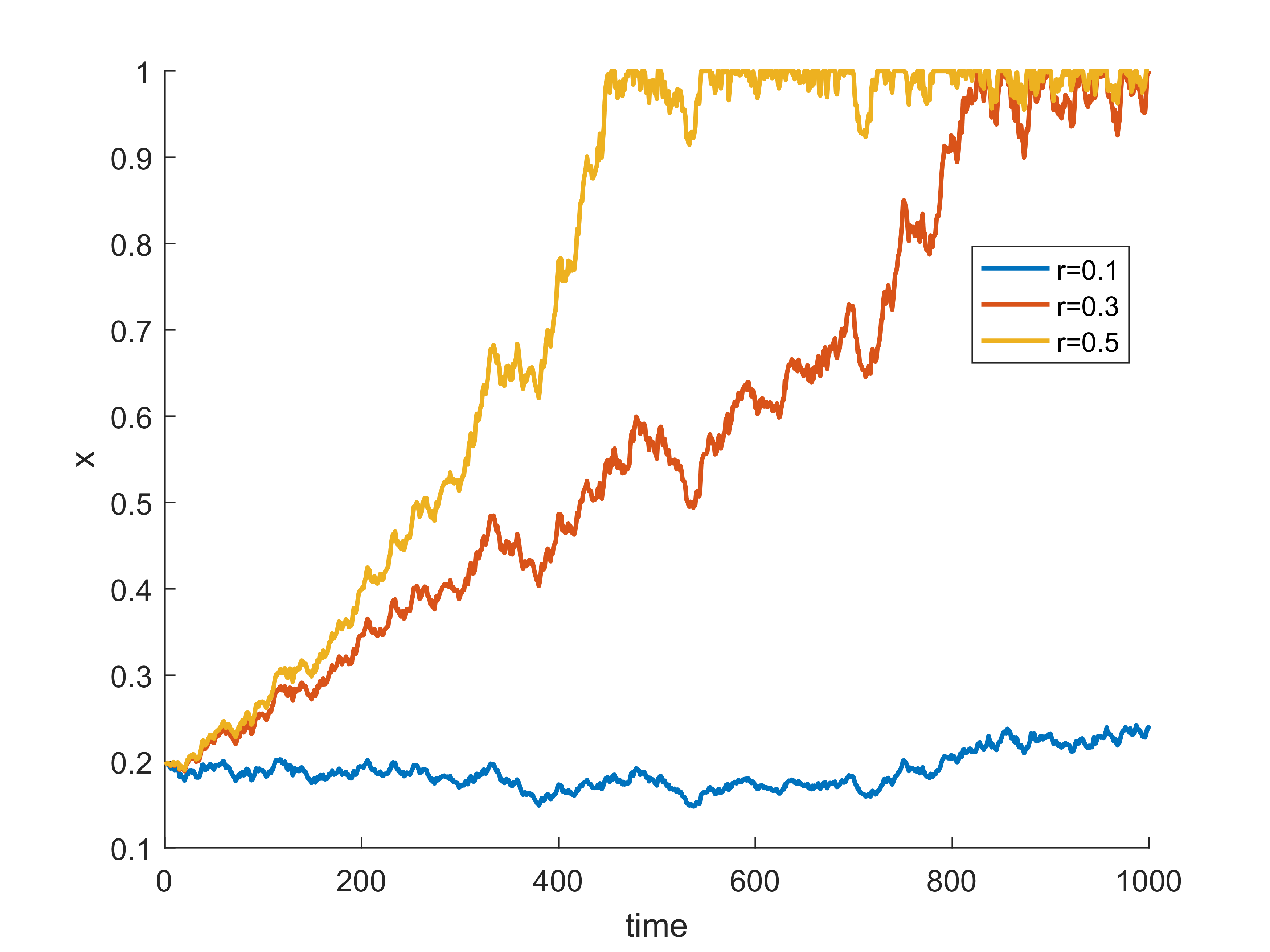


**S4 Fig. Impact of government digitalization efficacy coefficient.**

With other parameters remaining unchanged, we assume $s=0.2, 0.4, 0.6$. S5 Fig illustrates the evolutionary trend of local enterprises’ strategies under different intensities of enterprise digital transformation efficacy. As $s$ increases, the speed at which $y$ tends to 1 becomes faster, and the probability of local enterprises choosing positively hiring older adults rises accordingly.


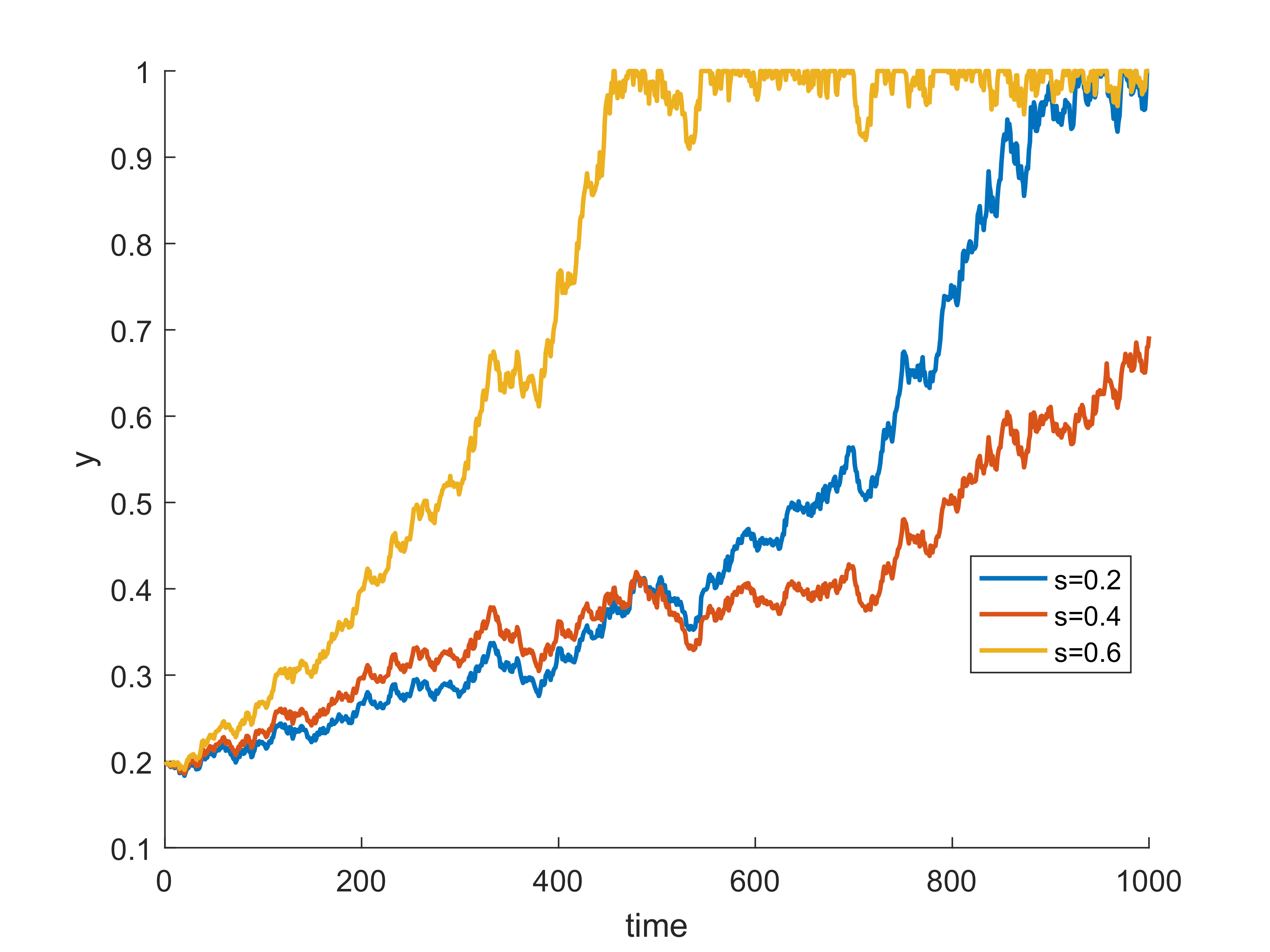


**S5 Fig. Impact of** **enterprise digital transformation efficacy coefficient.**

With other parameters remaining unchanged, we assume $R5=2, 4, 6$. S6 Fig illustrates the evolutionary trend of older adults’ strategies under different intensities of the benefit of labor participation. As $R5$ increases, the speed at which $z$ tends to 1 becomes faster, and the probability of older adults choose to participate in labor rises accordingly.


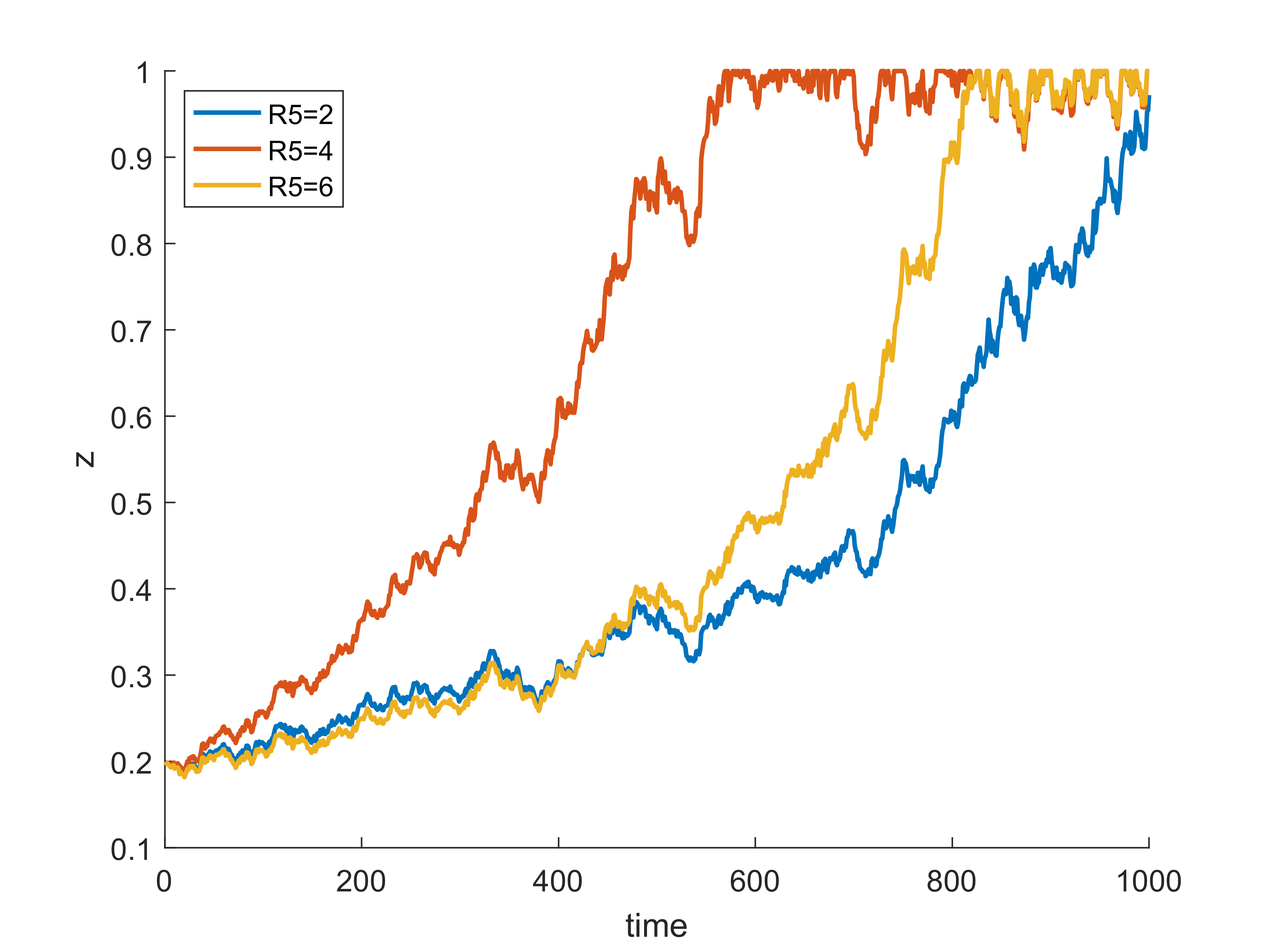


**S6 Fig. Impact of the benefit of labor participation.**

**References**

1. Baker CTH, Buckwar E. Exponential stability in p-th mean of solutions, and of convergent Euler-type solutions, of stochastic delay differential equations. Journal of Computational and Applied Mathematics. 2005;184(2):404–427. <https://doi.org/10.1016/j.cam.2005.01.018>
